# Unconventional codon usage bias mediates mRNA translational dynamics in macrophages

**DOI:** 10.1371/journal.pbio.3003403

**Published:** 2025-09-18

**Authors:** Shiqi Luo, Qiuyi Wang, Ao Chen, Liang Huang, Yaoqi Liu, Xin Zong, Yuanhui Mao

**Affiliations:** 1 Department of Urology of The Second Affiliated Hospital of Medicine & Liangzhu Laboratory, College of Animal Sciences, Zhejiang University, Hangzhou, China; 2 Key Laboratory of Molecular Animal Nutrition, Ministry of Education, Hangzhou, China; 3 Institute of Biochemistry, College of Life Sciences, Zhejiang University, Hangzhou, China; Johns Hopkins University, UNITED STATES OF AMERICA

## Abstract

Macrophages require rapid and tightly controlled regulatory mechanisms to respond to environmental disruptions. While transcriptional regulation has been well characterized, the mechanisms underlying translational control in macrophages remain poorly understood. Here, we investigated the dynamics of mRNA translation in mouse macrophages during acute, intermediate, and prolonged LPS exposure. Our results reveal clear phase-specific translational regulation during macrophage polarization, which initially increases the synthesis of inflammatory mediators and cytokines, while simultaneously suppressing the expression of cell cycle-related genes. Mechanistically, we observed pervasive upstream translation in the 5′ UTRs of cell cycle-related mRNAs, which contributes to cell cycle arrest during the early phase of inflammatory response. Notably, we identified a unique codon preference toward A/U in the third position of codons in macrophages, which contrasts with the G/C preference commonly observed in other tissues. AU codon preference increases the stability and translation efficiency of cell cycle-related mRNAs, promoting cell cycle restoration after extended LPS exposure. These findings reveal that uORF translation and codon usage bias are critical components of translational regulation during macrophage polarization, highlighting a potential therapeutic intervention for modulating immune activation via macrophage-specific codon optimization.

## Abstract 2

Macrophage polarization involves distinct phases of translational regulation that coordinate inflammatory responses. This study shows that upstream translation and AU-rich codon usage enhance mRNA stability and translation efficiency, enabling dynamic control of immune activation and cell cycle restoration during prolonged inflammatory stimulation.

## Introduction

mRNA translation in immune system is tightly orchestrated to enable a rapid response to diverse environmental stimuli [1–7]. Among the cellular components of the immune system, T cells are the most well-characterized examples where translational control plays a critical role in T-cell function [8–11]. For example, upon antigen recognition, naïve T cells differentiate into highly proliferative effector cells, accompanied by extensive changes in mRNA translation. During this process, the tRNA pool in T cells can rapidly shift, favoring the translation of A/U-rich codons, subsequently promoting the translation of genes involved in cell proliferation [11]. Characterizing the dynamic changes in mRNA translation and elucidating the underlying mechanisms is fundamental to understanding the role of the immune system in health and disease. Despite extensive studies on T cells, our understanding of translational control in other immune cells is not fully understood.

Macrophages are essential components of the innate immune system. Upon encountering bacterial pathogens, macrophages can polarize into either classically activated macrophages, known for their pro-inflammatory and antimicrobial functions [12,13], or alternatively activated macrophages [14,15], which might be involved in anti-inflammatory responses and tissue repair [16–18]. Multiple signaling pathways [19], such as the C-Jun N-terminal kinase (JNK) [20], PI3K/Akt [21,22], Notch signaling cascades [23], and the mammalian target of rapamycin (mTOR) pathway [24,25], play critical roles in driving dynamic changes in the expression of macrophages. Among them, mTOR activation is necessary for the metabolic reprogramming that enables biosynthetic support and regulatory control of macrophage polarization [24–28]. Notably, mTOR plays dual roles during macrophage polarization, by affecting both pro-inflammatory cytokines, such as IL-6 and TNF-α [29,30], as well as anti-inflammatory cytokines like IL-10 [31,32]. The timing and duration of mTOR activation at different phases of macrophage polarization are likely critical for the transition between pro-inflammatory and anti-inflammatory states. Given the central role of mTOR in regulating protein translation [33,34], translational control may serve as an additional layer in the regulation of macrophage polarization [35–37]. Previous studies have shown that global protein synthesis in macrophages increases dramatically within the first hour following lipopolysaccharide (LPS) stimulation [38,39], indicating substantial protein demand during the initial inflammatory response [2]. On the other hand, many feedback inhibitors of the inflammatory response, including NF-κB inhibitors, are subsequently upregulated, leading to a downregulation of inflammatory response genes and a restoration of global protein synthesis levels [39]. Moreover, previous studies using either ribosome profiling (Ribo-seq) or RNA-seq revealed “intensified” regulation at both the transcriptional [40] and translational [41] levels in response to LPS stimulation.

While previous studies have revealed differential translation in macrophages, the detailed mechanisms underlying translational dynamics remain largely unclear. Interestingly, previous studies revealed a critical role of codon usage in regulating mRNA translation, which in turn modulates cellular proliferation [42,43]. Synonymous codons, different codons that encode the same amino acid, are not used uniformly across coding sequences [44,45], known as codon usage bias. Codon usage bias is a key characteristic of coding regions and has dramatic effects on translation efficiency (TE) [46,47] and mRNA stability [48,49]. While previous studies revealed a distinct codon usage pattern in T cells [11], it remains unclear whether macrophage polarization is affected by codon usage-mediated mRNA translation and metabolism.

In this study, we characterized the dynamics of mRNA translation from the early phase to the late phase of macrophage polarization induced by LPS. Our study revealed notable time-dependent mRNA translation at different phases of macrophage polarization. We identified widespread upstream translation initiation, which contributes to the cell cycle arrest during the early phase of macrophage polarization. Furthermore, our results revealed a distinct codon usage pattern in mouse macrophages compared with other mouse tissues, suggesting a tissue-specific mechanism for regulating mRNA stability and TE during macrophage polarization.

## Results

### Characterization of time-dependent mRNA translation during macrophage polarization

**Fig 1:**
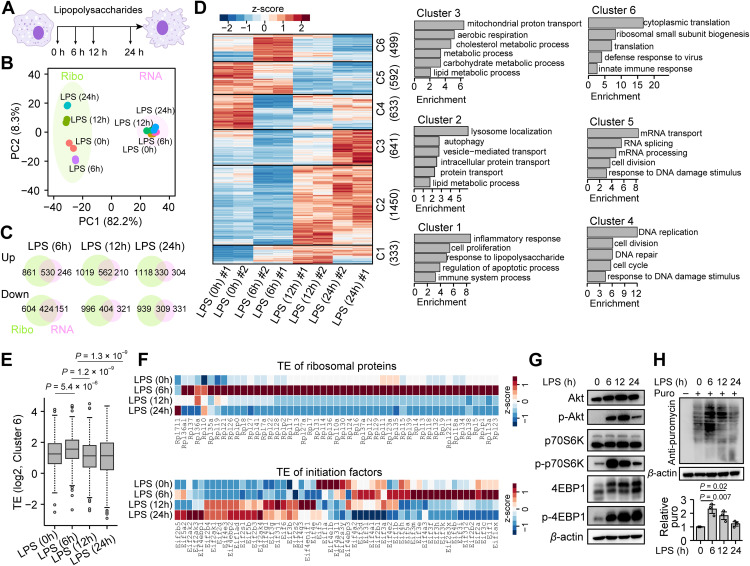
Landscape of translation dynamics during macrophage polarization. **(A)** Schematic representation of sample collection timepoints during macrophage polarization. **(B)** Principal component analysis (PCA) showing variation of ribosome density (Ribo-seq) and mRNA abundance (RNA-seq) at different timepoints during macrophage polarization, using the top 1,000 mRNAs with the highest ribosome densities. **(C)** Venn diagrams showing the number of differentially expressed mRNAs during macrophage polarization. **(D)** Heatmap showing ribosome densities for different clusters of differentially expressed mRNAs across timepoints during macrophage polarization. The bar plots on the right show the enriched biological processes for each cluster (C1–C6). To visualize the expression patterns of individual mRNAs in heatmaps, a z-score normalization across time points for each gene was applied using the eq. (1) in Methods. The original RPKM values were shown as boxplots in S3C Fig. **(E)** Boxplot showing the translation efficiencies of cluster 6 at different timepoints. Wilcoxon tests were performed between each pair of samples. For each gene, a mean value of the two biological replicates was used. **(F)** Heatmap of ribosome densities for ribosomal proteins and initiation factors across timepoints. The Z-score normalization method same as Fig 1D was performed. The original TE values were shown as a heatmap in S3D Fig. **(G)** A representative western blot of mTOR pathway-related proteins in macrophages before and after LPS treatment at the indicated timepoints. The quantification of the immunoblot data was shown as bar plots in S3E Fig. **(H)** Puromycin labeling assay in macrophages before and after LPS treatment. Bar plots show the relative levels of puromycin-labeled nascent chains quantified by densitometry. Error bars represent mean ± SEM, unpaired two-tailed *t* test, *n* = 3 biological replicates. The data underlying the graphs shown in the figure can be found in S1 Data. Raw blot images can be found in S1 Raw Images.

To investigate the dynamics of mRNA translation during macrophage polarization, we established a macrophage activation model using the mouse macrophage cell line Raw264.7 stimulated with LPS. We monitored mRNA translation and steady-state mRNA level using Ribo-seq and RNA-seq, respectively, at 4 time points over 24 hours (0, 6, 12, and 24 hours) of LPS treatment (Figs 1A and S1A). LPS treatment for 6 hours resulted in a dramatic increase in the mRNA levels of pro-inflammatory genes including tumor necrosis factor-alpha (*Tnf-α*), interleukin-6 (*Il-6*), and interleukin-1 beta (*Il-1β*) (S1B Fig), confirming macrophage activation. In addition, we observed a significant reduction of mRNA levels in *Il-1β* and *Tnf-α* levels after 24 hours of LPS treatment, suggesting a reduced pro-inflammatory response after long-term LPS stimulation [50]. To investigate translational control in macrophages, we adapted our previously established method [51] to construct the Ribo-seq libraries in this study (see Method for details). As a result, the ribosome footprint length across all eight Ribo-seq samples was ~29 nt (S1C Fig), consistent with the expected size of ribosome-protected fragments in mammalian cells [52]. Furthermore, ~85% of the reads were mapped to coding region (S1D Fig), and more than 70% of the reads were assigned to the correct reading frame (S2 Fig). These results indicate the high-quality of Ribo-seq dataset in this study.

We subsequently performed principal component analysis (PCA) to quantify the dynamic changes in both mRNA steady-state abundance and ribosome density. As expected, LPS treatment altered the landscape of gene expression in macrophages at both transcriptional and translational levels (S3 Fig). A previous study based on proteomics in dendritic cells revealed that translational control contributes more than transcriptional control to changes in protein expression, particularly for housekeeping genes [38]. We therefore focused on the high-abundance mRNAs in macrophages. Interestingly, we observed notable changes in ribosome density after 6 hours of LPS treatment, with differences being more pronounced after 12 and 24 hours (Fig 1B). In contrast, those high-abundance mRNAs show fewer changes in mRNA levels upon LPS treatment. In addition, differential expression analysis identified thousands of genes whose expression was altered at either the level of ribosome density or mRNA abundance, with the number of differentially expressed genes at the ribosome density being almost 2-fold higher compared to mRNA abundance level (Fig 1C). These data suggest an important role of translational regulation during macrophage polarization, especially for high-abundance mRNAs.

We then performed analysis of variance (ANOVA) to identify uniquely expressed genes at each time point, which revealed a clear time-dependent expression of “marker genes”. After 6 hours of LPS treatment, we observed a significant upregulation in the ribosome density of genes involved in the inflammatory response (Fig 1D, Cluster 1), which becomes even higher after 12 hours of LPS treatment. Interestingly, the ribosome density of inflammatory response genes after 24 hours of LPS treatment is decreased, compared to 12 hours of LPS stimulation. This result is further supported by our qPCR analysis of individual genes (S1B Fig), suggesting a likely transition towards the resolution of inflammation or inflammation tolerance following prolonged LPS stimulation. Moreover, we observed a notable increase in the ribosome density of mRNA involved in protein translation after 6 hours of LPS treatment (Fig 1D, Cluster 6), suggesting enhanced protein synthesis. Unlike inflammatory response genes, the elevated ribosome density of translation-related mRNAs was reduced after 12 hours of LPS treatment. This pattern is consistent with the substantial production of cytokines and chemokines during the early phase of the inflammatory response [53,54]. Interestingly, genes involved in protein transport were also upregulated after 12 hours or 24 hours of LPS treatment (Fig 1D, Cluster 2), which suggests a possible coordination between the synthesis and secretion of cytokines and chemokines.

Ribosome density on mRNAs typically correlates with mRNA abundance. Indeed, we observed a good correlation between changes in ribosome density and mRNA levels across most identified gene clusters (S3B and S3C Fig). However, when mRNA abundance was corrected, we still observed a significant increase in TE in translation-related mRNAs after 6 hours of LPS treatment (Fig 1E), followed by a reduction after 12 or 24 hours of LPS treatment. Consistent with this observation, the expression of most ribosomal proteins is increased, whereas translation initiation factors remained unchanged (Figs 1F and S3D). Because most ribosomal proteins contain a 5′ terminal oligopyrimidine tract (TOP) or TOP-like sequence in their 5′ UTR, which is sensitive to the activity of mTOR complex 1 (mTORC1) [34]. Therefore, the increase in TE of ribosomal protein mRNAs after LPS treatment is likely due to an increase in mTORC1 activity. In line with this hypothesis, we observed that 6 hours of LPS treatment leads to increased phosphorylation of Akt and the mTORC1 downstream targets p70S6K and 4EBP1 (Figs 1G and S3E). To further investigate whether global translation increases in parallel with elevated ribosomal protein levels, we examined endogenous protein synthesis using puromycin labeling. As a result, we observed a significant increase in protein synthesis after 6 hours of LPS treatment (Fig 1H), which was subsequently reduced after 12 and 24 hours of LPS treatment. We further validated this finding in mouse bone marrow-derived macrophages (BMDMs), which exhibited a similar pattern (S4 Fig). These results suggest transient activation of mRNA translation during the early phase of the inflammatory response.

### Translational regulation contributes to the arrest of cell cycle during macrophage polarization

**Fig 2:**
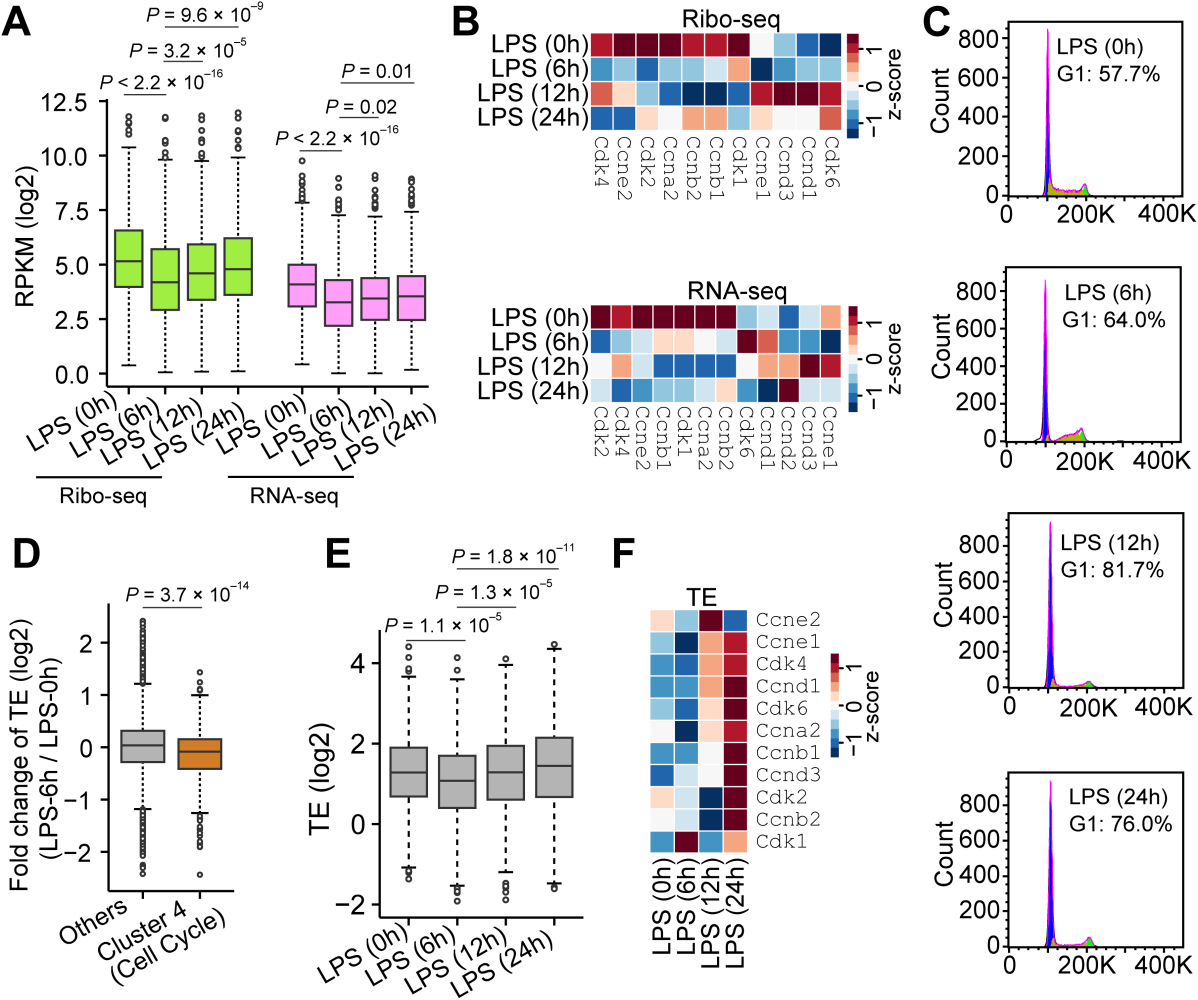
Translation regulation of cell cycle-related genes during macrophage polarization. **(A)** Boxplot showing dynamic changes of ribosome density (Ribo-seq) and mRNA abundance (RNA-seq) of cell cycle-related genes during macrophage polarization. Wilcoxon tests were performed between each pair of samples. For each gene, a mean value of the two biological replicates was used in all analyses unless otherwise indicated. **(B)** Heatmaps showing changes in the ribosome density (Ribo-seq) and mRNA abundance (RNA-seq) of individual cell cycle-related mRNAs. Z-score normalization same as Fig 1D was performed. The original values were shown as a heatmap in S5B Fig. **(C)** Flow cytometry analysis showing cell cycle arrest during macrophage polarization. **(D)** Boxplot showing fold change in translation efficiency (TE) (Ribo-seq normalized by RNA-seq) after 6 hours of LPS treatment compared to control cells. mRNAs in macrophages were separated into two groups: cluster 4 (same as Fig 1D) and others. Wilcoxon tests were performed between the two groups. **(E)** Boxplots showing dynamic changes in TE of cluster 4 (cell cycle-related) at different timepoints during macrophage polarization. Wilcoxon tests were performed between each pair of samples. **(F)** Heatmaps showing changes in TE for individual cell cycle-related mRNAs. Z-score normalization same as Fig 1D was performed. The original values were shown as a heatmap in S5B Fig. The data underlying the graphs shown in the figure can be found in S1 Data. Representative raw flow cytometry plots showing the gating strategy can be found in S1 Raw Images.

Despite an overall increase in the global translation level via mTORC1 activation (Fig 1G and 1H), we unexpectedly observed significant downregulation of genes involved in the cell cycle after 6 hours of LPS treatment (Fig 1D, Cluster 4). A closer examination of genes in cluster 4 (cell cycle-related genes) revealed a decrease in both ribosome density and mRNA abundance within 6 hours (Fig 2A). Furthermore, the master genes, which control the transition of the cell cycle at different checkpoints, show a consistent reduction in 6 hours (Figs 2B, S5A, and S5B), suggesting potential arrest of the cell cycle during the early phase of inflammation. Indeed, when analyzing the cell cycle using flow cytometry, we observed a clear increase in the cell population arrested at the G1 stage and a concomitant reduction in the S1 and G2 stages (Figs 2C and S5C).

As a fundamental biological process, the cell cycle is tightly orchestrated and involves a series of ordered and controlled events [55]. Cell cycle progression requires not only a substantial amount of cell mass but also a favorable nutritional status [56]. The mTOR signaling pathway regulates cell cycle progression by coordinating cellular growth with nutritional cues [57]. Therefore, mTOR activation by pathogens could send conflicting signals regarding cell cycle progression in macrophages, potentially inducing apoptosis [58,59]. To investigate the role of mRNA translation in cell cycle arrest under mTOR activation, we analyzed the TE of cell cycle-related mRNAs by normalizing ribosome density to the corresponding mRNA abundance, which revealed a significant reduction in TE after 6 hours of LPS treatment (Fig 2D). Intriguingly, the TE is recovered to the normal level (Fig 2E), whereas the transcription level remains suppressed even after 24 hours of LPS treatment (Fig 2A). Further analysis of individual cell cycle checkpoint genes, including *Cdk2*, *Cdk4*, and *Cdk6*, confirmed a similar reduction in TE after 6 hours of LPS treatment (Fig 2F). We further confirmed these results using the mouse BMDMs stimulated with LPS [60], which again showed a reduction in the expression of cell cycle-related mRNAs after 6 hours of LPS treatment (S5D Fig).

### Upstream translation in 5′ UTR reduces the expression of cell cycle-related mRNAs

**Fig 3:**
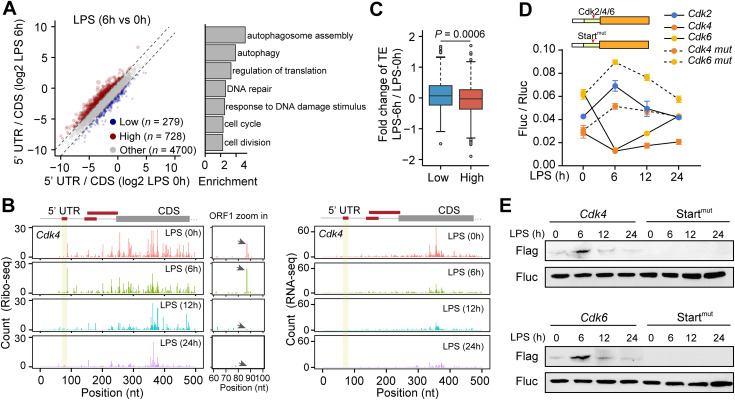
Upstream translation regulates the translation of cell cycle-related genes. **(A)** Scatter plot showing the ratio of Ribo-seq reads in 5′ UTR over the reads in CDS. An increasing ratio suggests an increase in upstream translation. The bar plot shows enriched biological processes for mRNAs with increased upstream translation (highlighted in red on the scatter plot). **(B)** Ribo-seq (left panel) and RNA-seq (right panel) reads of *Cdk4* mRNA. The upstream open reading frames detected by Ribo-seq are highlighted by red frames. The right zoom-in panel showing ribosome density on uORF1 of *Cdk4* mRNA, the position of stop codon of uORF1 is indicated by arrows. **(C)** Boxplot showing the inverse relationship between changes in upstream translation and translation efficiency (TE) in main CDS. “High” refers to mRNAs with the highest upstream translation (top 30%), and “Low” refers to mRNAs with the lowest upstream translation (bottom 30%). Wilcoxon tests were performed between the two groups. **(D)** Reporter assay showing TE when the 5′ UTR sequence of *Cdk2*, *Cdk4*, or *Cdk6* was inserted into the 5′ UTR of a firefly luciferase reporter. Mutant constructs in which the start codon of the uORF in *Cdk4* or *Cdk6* was changed to a nonstart codon (*Cdk4-mut* and *Cdk6-mut*) were also generated. Renilla luciferase was co-transfected with the firefly luciferase constructs and served as an internal control (*n* = 3 biological replicates). **(E)** Western blot showing translation of uORF in *Cdk4* or *Cdk6* 5′ UTR. A 3× FLAG tag was inserted immediately upstream of the stop codon of uORF1 of the *Cdk4* mRNA, or uORF of the *Cdk6* mRNA. The expression of the uORF was detected via immunoblotting analysis. Start^mut^ refers to the mutant reporters where the start codon of uORF was mutated to a nonstart codon. The expression of firefly luciferase was used as a control for difference in transfection efficiency and cell numbers. The data underlying the graphs shown in the figure can be found in S1 Data. Raw blot images can be found in S1 Raw Images.

To investigate the mechanisms underlying the translational suppression of cell cycle-related mRNAs during macrophage polarization, we analyzed the sequence characteristics of these mRNAs. Interestingly, while cell cycle-related mRNAs exhibit 5′ UTR lengths similar to those of other genes, they tend to have higher GC contents and more RNA secondary structures in the 5′ UTR (S6A–S6C Fig), implying a potential role of the RNA secondary structure in regulating the translation of cell cycle-related mRNAs. RNA secondary structures in the 5′ UTR can impair ribosomal scanning, thus increasing the probability of initiation within the 5′ UTR [3,61–63]. We therefore focused our analysis on translation initiation within the 5′ UTR, which revealed a significant increase in ribosome density within the 5′ UTR after 6 hours of LPS treatment (Fig 3A), indicating enhanced upstream initiation during macrophage polarization. Notably, mRNAs with increased upstream initiation were enriched in the cell cycle and autophagy processes, suggesting a potential role of upstream initiation in translational regulation of cell cycle-related mRNAs. We further validated this result by calculating the initiation potential index in 5′ UTR, which we previously used to estimate the frequency of initiation at all potential initiation sites in 5′ UTR [51]. As a result, the mRNAs with high initiation potential in the 5′ UTR were again enriched in cell cycle-related processes (S6D Fig).

To decipher the role of uORFs in cell cycle regulation, we subsequently identified upstream open reading frames (uORFs) at different time points upon LPS treatment. Interestingly, we detected three uORFs in *Cdk4* mRNA (Fig 3B) and one uORF in *Cdk6* mRNA (S1 Table). Notably, uORF translation in the *Cdk4* mRNAs became apparent in the samples with 6 hours of LPS treatment, but was markedly reduced or absent in samples treated for 12 or 24 hours (Fig 3B). uORFs can reduce the translation of the main coding sequence (CDS) by attenuating ribosome scanning [64,65]. Indeed, we observed a negative correlation between mRNAs with upstream ribosome density and downstream ribosome density (Fig 3C). While Ribo-seq is sensitive enough to detect uORFs with robust translation, read counts at individual nucleotide positions are typically sparse, making it challenging to examine uORF dynamics on individual mRNAs. To address this, we inserted the 5′ UTR of *Cdk4* or *Cdk6* into the upstream region of a firefly luciferase reporter. In addition, we also inserted the 5′ UTR of *Cdk2*, which lacks a detectable uORF, into the luciferase reporter. A Renilla luciferase reporter, serving as an internal control, and the firefly luciferase reporter were co-transfected into Raw264.7 cells. We observed a gradual increase in activity of the luciferase reporter containing a *Cdk2* 5′ UTR, consistent with the dynamic changes of global translation upon LPS treatment (Fig 1H). However, luciferase activity was significantly reduced after 6 hours when either a *Cdk4* or *Cdk6* 5′ UTR was inserted, with translation gradually recovering after 12 and 24 hours (Fig 3D). Notably, the mutant constructs with the uORF start codon changed to a nonstart codon exhibited increased reporter activity compared to the wild-type reporter (Fig 3D). In addition, we assessed the uORF translation by inserting a 3× FLAG tag immediately upstream of the stop codon of uORF. Western blot analysis revealed an increased level of the FLAG-tagged peptide after 6 hours of LPS treatment (Fig 3E), indicating enhanced uORF translation at this time point. Overall, these results suggest that upstream translation in cell cycle-related mRNAs reduces the translation of their main CDS, contributing to cell cycle arrest in the early phase of macrophage polarization.

### Dynamic shifts of codon usage affect TE of cell cycle-related genes

**Fig 4:**
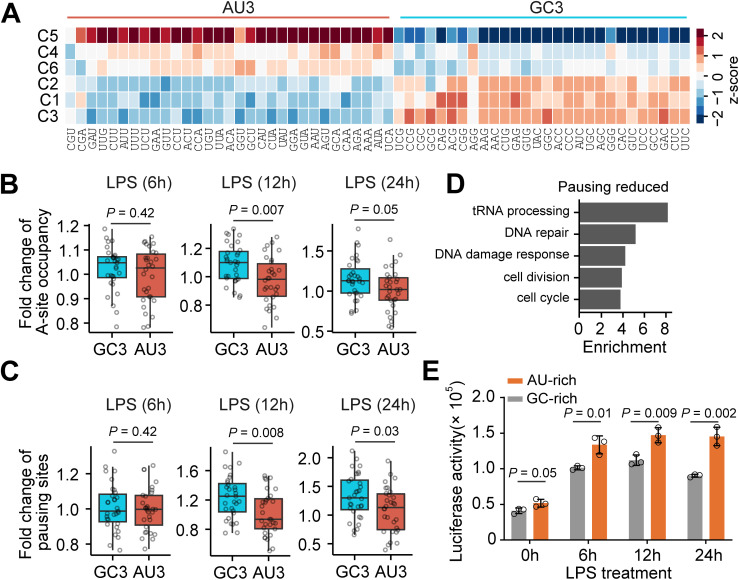
Dynamic changes in ribosomal pausing. **(A)** Heatmap showing dynamic changes in codon usage bias across different gene clusters (C1–C5) as defined in Fig 1D. Codon usage bias was quantified by the relative synonymous codon usage (RSCU) values. To visualize RSCU values across different clusters, z-score normalization for each cluster was applied using the eq. (1) in Methods. As a result, each rows have the same mean value of 0 and standard deviation of 1. **(B)** Boxplot showing fold changes in ribosomal occupancy at individual codons at the ribosomal A-site by comparing cells with (6, 12, or 24 hours) or without LPS treatment. Wilcoxon tests were performed between each pair of samples. **(C)** Boxplot showing fold changes of ribosomal pausing sites at individual codons by comparing cells with (6, 12, or 24 hours) or without LPS treatment. Wilcoxon tests were performed between each pair of samples. **(D)** GO analysis showing enriched biological processes for mRNAs with reduced ribosomal pausing sites. **(E)** Bar plot showing firefly luciferase activity when a wildtype (GC-rich) or a mutant (AU-rich) luciferase reporter was transduced into cells. Error bars represent mean ± SEM, unpaired two tailed *t* test, *n* = 3 biological replicates. The data underlying the graphs shown in the figure can be found in S1 Data.

By analyzing TE of cell cycle-related mRNAs, we observed a gradual recovery of TE after 12 hours of LPS treatment (Figs 2E and 3D), which could be partially due to reduced upstream initiation (Fig 3B). Intriguingly, our reporter assay also showed that the translation of *Cdk2* mRNA remained elevated, although the global translation returned to normal level after 24 hours (Fig 1H). These data suggest additional mechanisms contributing the translational dynamics of cell cycle-related mRNAs. Numerous studies have demonstrated that codon usage bias is another major factor influencing TE (reviewed in [47]). We therefore analyzed codon usage bias across different gene clusters shown in Fig 1D, which uncovered distinct codon usage patterns (Fig 4A). The genes related to inflammatory response, protein transport, and metabolic processes exhibit a strong bias toward codons with G/C at the third position (GC3). In contrast, genes related mRNA translation and cell cycle showed a relative preference for codons with A/U at the third position (AU3). Notably, codon usage bias is not affected by amino acid frequency difference in different clusters, since we calculated the relative codon usage normalized by amino acid frequency (relative synonymous codon usage, RSCU [45,66]).

Previous studies have reported that mRNAs enriched with AU3 codons are translated more efficiently in proliferating cells [43,67]. Since different gene clusters shown in Fig 1D were expressed in a time-dependent manner, the codon usage bias across gene clusters suggests a potential dynamic shift of codon usage during the different phases of macrophage polarization. To investigate whether TE is affected by codon usage dynamics, we calculated ribosome occupancy at all 61 individual codons. Notably, we observed a significant reduction of ribosome occupancy on AU3 codons after 12 or 24 hours of LPS treatment (Fig 4B), indicating an increase in translation elongation rate on AU3 codons. To further validate this result, we identified ribosomal pausing sites on individual mRNAs, which revealed a significant increase in ribosomal pausing sites on GC3 codons after 12 or 24 hours of LPS treatment (Fig 4C). Interestingly, GO analysis showed that mRNAs with reduced ribosomal pausing sites are enriched in the cell cycle-related process (Fig 4D). These results suggest that cell cycle-related mRNAs enriched with AU3 codons tend to be translated more efficiently during macrophage polarization after middle- or long-term LPS stimulation.

To further confirm that mRNA translation is enhanced by AU3 codons, we constructed a luciferase reporter by inserting an *N*-terminal 3× FLAG sequence enriched with GC3 codons (GC-rich). A synonymous mutant reporter was generated by replacing GC3 codons with AU3 codons, without altering the amino acid sequence (AU-rich). Both reporters were independently transduced into Raw264.7 cells, and luciferase activity was measured before and after LPS treatment. Consistent with Ribo-seq analysis, the AU-rich reporter exhibited significantly higher protein level than the GC-rich reporter, with the difference being most pronounced after 24 hours of LPS treatment (Fig 4E). In addition, we reanalyzed publicly available proteomics datasets from LPS-treated mouse BMDMs [68], which again revealed significantly enrichment of AU3 codons in mRNAs with increased protein levels after 24 hours of LPS treatment (S7A Fig). In addition to higher expression, the AU-rich reporter displayed a marked increase in mRNA stability after 6, 12, and 24 hours of LPS treatment, whereas the GC-rich reporter showed only a modest change (S7B and S7C Fig). Previous studies have found that codon optimality is a key determinant of mRNA stability in mammalian cells [48,49]. Thus, the enhanced stability of the AU-rich construct supports a shift of codon optimality toward AU-rich codons after LPS treatment. Taken together, these results support a model in which AU-rich codons contribute to the translational reprogramming observed during macrophage polarization.

### Codon usage-dependent RNA stability regulates the recovery of cell cycle-related gene expression

**Fig 5:**
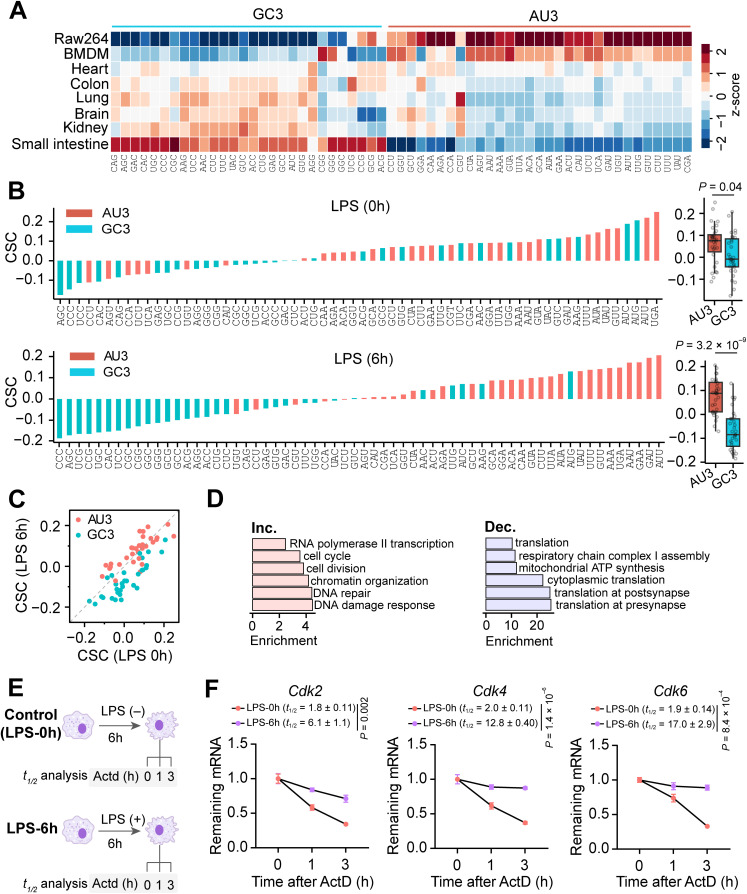
Dynamic changes in mRNA stability. **(A)** Codon usage bias of housekeeping genes across different mouse tissues. To visualize RSCU values across different clusters, a z-score normalization for cell lines or tissues was applied using the eq. (1) in Methods. As a result, each rows have the same mean value of 0 and standard deviation of 1. BMDM: mouse bone marrow-derived macrophage. **(B)** Correlation between mRNA stability and codon frequency, represented by codon stabilization coefficient (CSC). Positive CSC values indicate codons that stabilize mRNAs, whereas negative values indicate codons that destabilize mRNAs. **(C)** A scatter plot showing CSC between LPS 0 hour and LPS 6 hour. A significant increase of CSC values for AU3 codon was observed in LPS 6 hours, compared with LPS 0 hour, indicating that AU3 codon further stabilizes mRNAs after 6 hours of LPS treatment. **(D)** GO analysis showing the enriched biological processes for mRNAs with increased (left) or decreased (right) stability in 6 hours of LPS treatment. **(E)** Schematic representation of sample collection time points for mRNA half-life analysis. **(F)** Comparison of mRNA stability in cells with (6 hours) and without LPS treatment. Left: schematic representation of the experimental workflow, including LPS and time points of ActD treatment for sample collection. Line plots showing relative mRNA levels after ActD treatment. Half-life values (*t*_*1/2*_) for each LPS treated samples were estimated using a linear regression (see Methods). Unpaired two tailed *t* test was performed to determine the statistical significance between half-life values of the two samples (*n* = 3, the values in parentheses represent the half-life values ± SD). Twelve or 24 hours of LPS treatment was shown in S8C Fig. The data underlying the graphs shown in the figure can be found in S1 Data.

Mammalian cells favor GC3 codons in most protein coding sequences [69,70]. Intriguingly, when analyzing codon usage at different phases of macrophage polarization, we found that the high-abundance mRNAs in macrophages were significantly biased toward AU3 codons, suggesting that the transcriptome of macrophages could have a distinct codon usage bias from that of other cells. To further explore this possibility, we compared codon usage of the high-abundance mRNAs across mouse tissues, which revealed a clear preference of AU3 codons in macrophages (Fig 5A). In contrast, in all other mouse tissues analyzed in this study, the high-abundance mRNAs were biased toward GC3 codons.

mRNA stability is affected by codon usage via regulation of the translation elongation rate [48,49]. In human cells, GC3 codons have been shown to stabilize mRNAs [71]. We validated this association in other cell lines, including mouse embryonic stem cells and embryoid body cells, using public datasets [72]. Interestingly, the results revealed a cell type-specific effect of codon usage on mRNA stability (S8A and S8B Fig). Having found a shift in the stability of reporter mRNAs (S7 Fig), we next investigated the association between codon usage and mRNA stability in the transcriptome of macrophages. Notably, our analysis revealed that AU3 codons in macrophages, rather than GC3 codons, stabilize mRNAs (Fig 5B), echoing AU3 codon preference in macrophages. We next investigated whether mRNA stability is affected by dynamic codon usage during macrophage polarization. Strikingly, we found that mRNAs enriched in AU3 codons showed increased stability after 6 hours of LPS treatment (Fig 5B and 5C). Moreover, mRNAs with increased stability were enriched in cell cycle-related process (Fig 5D). In contrast, the mRNAs with reduced stability were enriched in translation and mitochondrial ATP synthesis. These data suggest that mRNA turnover is associated with dynamic changes of codon usage during macrophage polarization. In macrophages, the increased degradation rate of translation-related mRNAs after 6 hours of LPS treatment could imply fast turnover of these mRNAs and a reduction in the global translation level after middle- or long-term LPS treatment (Fig 1F and 1G). In contrast, although translation of the cell cycle-related mRNAs was decreased after 6 hours of LPS treatment, the mRNAs remained stable, which promoted the rapid expression of cell cycle-related mRNAs after middle- or long-term LPS treatment using the preexisting mRNAs. We further validated this result by estimating the half-life of endogenous cell cycle-related genes *Cdk2*, *Cdk4*, and *Cdk6* via qPCR analysis (Fig 5E), which revealed a significantly increased mRNA half-life after LPS treatment (Figs 5F and S8C). Taken together, these data uncovered a codon usage-associated regulation on mRNA stability, which could suggest a rapid adaptive response to environmental perturbations in a posttranscriptional manner.

## Discussions

**Fig 6:**
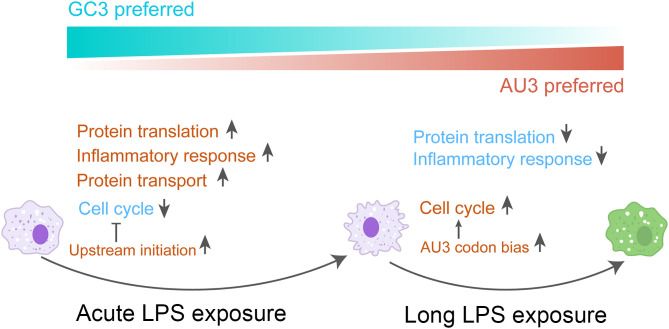
Translational regulation during macrophage polarization. LPS stimulation induces time-dependent translational regulation, which is associated with uniquely dynamic changes of codon usage in macrophages.

Macrophages require precise regulatory mechanisms to respond rapidly to environmental perturbations while maintaining homeostasis to prevent excessive inflammatory responses. Protein translation regulation provides a rapid and reversible way to enable macrophages to produce necessary inflammatory mediators using preexisting mRNAs. In this study, our comprehensive analysis of translational regulation during acute, intermediate, and long-term LPS exposure revealed sophisticated translational control to orchestrate the inflammatory response (Fig 6).

### Translational regulation across phases of inflammation

During the initial phase of polarization, macrophages exhibit a dramatic increase in translational activity through mTOR pathway activation, supporting the substantial demand for inflammatory mediators and cytokines (>1,000-fold increase in inflammation-related proteins). Concurrent with elevated cytokine production, we observed upregulation of protein transport machinery, suggesting coordinated adaptation of multiple pathways during the inflammatory response. In addition, both the transcription and translation of cell cycle-related genes are suppressed during early polarization, which may represent a protective mechanism against apoptosis when mTOR is activated by inflammatory signals rather than nutritional signals [73]. Notably, we identified pervasive upstream translation in the 5′ UTRs of cell cycle-related genes, including the checkpoint genes *Cdk4* and *Cdk6*, which contribute to cell cycle arrest during the early phase of inflammatory response.

After prolonged LPS exposure, macrophages tend toward homeostatic recovery, characterized by attenuated translational activity and inflammatory responses, accompanied by cell cycle restoration. Interestingly, we observed a dynamic shift of codon usage that supports cell cycle restoration. Cell cycle-related mRNAs are enriched in AU3 codons. After prolonged LPS exposure, the AU3 codons are translated more efficiently than GC3 codons, which thus promotes TE of cell cycle-related mRNAs. Furthermore, mRNAs enriched in AU3 codons become more stable over time, which further enhances the rapid expression of cell cycle-related proteins during recovery.

### Unique characteristics of codon usage in macrophages

Perhaps the most unexpected finding is a phase-specific shift in codon usage during macrophage polarization. We found that mouse macrophages exhibit a unique bias for AU3 codons, in contrast to the GC3 preference observed in other mouse tissues [69,70]. This distinctive codon usage pattern indicates a tissue-specific adaptation of protein synthesis. Intriguingly, the preference for AU3 codons in macrophages resembles the codon usage bias observed in many bacteria, which suggests a possible coevolutionary relationship between the innate immune system and bacterial organisms [74,75]. Using small RNA-seq, we found that the tRNA pool in macrophages was still biased toward GC3 codons (S9 Fig), consistent with observations in other cell types, indicating that AU3 codons in macrophages are relatively nonoptimal under normal condition. Therefore, AU3 codon usage bias in macrophages could suggest that many highly abundant mRNAs (e.g., cell cycle-related mRNAs) remain translationally inactive despite higher basal mRNA levels. Upon prolonged LPS exposure, translation of AU3 codons becomes more efficient than that of GC3 codons, suggesting a shift of tRNA pool from GC3 bias to AU3 bias, a phenomenon similar to the dynamic changes of tRNA pool observed during T-cell activation [11].

### Therapeutic implications of codon usage in macrophages

Understanding the unique features of codon usage and its dynamics in macrophages during inflammation has significant therapeutic potential. Mammalian genomes are well known biased toward GC3 codons, and GC3 codons in many human and mouse source cell lines are more optimal than AU3 codons [76]. Therefore, when designing an mRNA sequence for a vaccine or medicine, most strategies tend to increase GC content (more GC3 codons) [77,78]. However, our findings on the dynamics of codon usage bias and the unique AU3 codon usage bias in macrophages highlight a cell type-specific optimization when designing an mRNA sequence.

## Methods

### Cell culture and reagents

RAW264.7 cells were cultured in 90% DMEM (C11995500BT, Gibco) supplemented with 10% FBS (FSP500, ExCell Bio). Human HEK293T cells were cultured in 90% DMEM (C11995500BT, Gibco) supplemented with 10% FBS (FSP500, ExCell Bio), 10,000 units/mL penicillin and 10,000 μg/mL streptomycin (10378016, Gibco). LPS (L5293, Sigma-Aldrich) was used to construct the macrophage inflammation model. Puromycin (A610593-0025, Sangon Biotech) was used for puromycin labeling and cell line construction. Actinomycin D (HY-17559, MedChem Express) was used for endogenous mRNA stability analysis. Antibodies used in this study were listed below: Akt (4685S, Cell Signaling Technology, 1:1000 WB), Phospho-Akt (4060S, Cell Signaling Technology, 1:1000 WB), p70 S6 Kinase (9202S, Cell Signaling Technology, 1:1000 WB), Phospho-p70 S6 Kinase (9205S, Cell Signaling Technology, 1:1000 WB), 4E-BP1 (9644T, Cell Signaling Technology, 1:1000 WB), Phospho-4E-BP1 (9451T, Cell Signaling Technology, 1:1000 WB), Puromycin (MABE343, Sigma-Aldrich, 1:5000 WB), DYKDDDDK Tag (8146S, Cell Signaling Technology, 1:800 WB), Firefly luciferase (ab185924, Abcam, 1:1000 WB), and β-actin (BS6007MH, Bioworlde, 1:2000 WB).

### Bone marrow-derived macrophages (BMDMs) isolation and culture

Eight-week-old female mice with a C57BL/6J background were purchased from Model Organisms (Shanghai, China). The protocols were approved by the Zhejiang University Institutional Animal Care and Use Committee (ZJU20241086). Bone marrow flushed from femurs and tibias was plated in 90% RPMI-1640 (C11875500BT, Gibco) supplemented with 10% heat-inactivated FBS (FSP500, ExCell Bio). Macrophage colony-stimulating factor (R&D Systems, 416-ML) was added at a final concentration of 5 ng/ml to obtain differentiated macrophages every 2 days for 6 days.

### Plasmid construction

The pLVX-puro vector was double-digested with EcoRI (R3101L, New England Biolabs) and NdeI (R0111V, New England Biolabs). A CMV fragment without the T7 promoter was amplified by PCR. The amplified fragment was then seamlessly cloned into the digested vector. Subsequently, a Kozak-Start-Luciferase fragment was inserted into the XhoI (R0146S, New England Biolabs) and XbaI (R0145S, New England Biolabs) sites of the modified vector, which was named pLVX-Kozak-puro. The 5′ UTRs of *Cdk2*, *Cdk4*, and *Cdk6* genes were synthesized by GenScript, and cloned into the pLVX-Kozak-puro vector between EcoRI and XbaI. The 3× FLAG fragment and the mutant fragment, in which the third bases of codons in 3× FLAG were synonymously mutated to A or T, were cloned together with the downstream luciferase fragments into the pLVX-Kozak-puro vector between XhoI and XbaI. The primers were listed in S1 Table.

### Cell line construction

HEK293T cells were seeded in 6-well plates and cultured in DMEM supplemented with 10% FBS at 37 °C with 5% CO₂. The four plasmids for lentiviral packaging were transfected into 293T cells using Lipo8000 Transfection Reagent (C0533, Beyotime) according to the manufacturer’s protocol. At 48- or 72-hours post-transfection, the virus-containing supernatant was collected. The supernatant was centrifuged at 3,000 rpm for 10 min to remove cell debris, and then filtered through a 0.45 μm filter (FF375, Beyotime). Genemore viral concentration kit (GM-040801, Genomeditech) was used to improve virus infection efficiency. RAW264.7 cells were seeded in 6-well plates and allowed to adhere overnight. One aliquot of the concentrated virus was used to infect RAW264.7 cells in the presence of polybrene (8 μg/ml) (C0351, Beyotime). After 24 hours, a second aliquot of the virus was added to the cells. At 48 hours postinfection, the medium was replaced with complete medium containing 2 μg/mL puromycin. The cells were selected with puromycin for 3 days. Surviving cells were expanded and used for further experiments.

### Luciferase assay

The constructed WT and Mut cell lines were seeded in 24-well plates, and stimulated with LPS for 6, 12, or 24 hours. The cells were washed twice with PBS, and then assayed for luciferase activity using a luciferase assay kit (E1500, Promega) according to the manufacturer’s protocol. Luminescence was quantified using a microplate reader (BMG LABTECH).

### RNA extraction and qPCR

Total RNA was extracted from cells using TRIzol LS (10296028CN, Thermo Fisher Scientific), and 500 ng of RNA was reverse-transcribed using HiScript III RT SuperMix for qPCR (+gDNA wiper) (R323, Vazyme). The resulting cDNA was diluted with an equal volume of water and used for qPCR on a CFX Duet Real-Time PCR System (Bio-Rad), using Taq Pro Universal SYBR qPCR Master Mix (Q712, Vazyme). Primers for each gene were listed in S2 Table.

### Immunoblotting

Cell lysates were prepared in RIPA buffer (P0013C, Beyotime) supplemented with protease (K1007, APExBIO) and phosphatase inhibitor cocktail (K1015, APExBIO). After incubating on ice for 15 min, the lysates were heated for 10 min, 100 °C in SDS-polyacrylamide gel electrophoresis (SDS-PAGE) sample buffer (P0286, Beyotime). Proteins were separated on a 10% SDS-polyacrylamide gel and transferred to a 0.2 μm Immobilon-PSQ (ISEQ00010, Merck Millipore). The membranes were blocked by 5% nonfat dry milk in TBS-T (TBS containing 0.1% Tween 20), and then blotted with primary antibodies overnight. After incubation with HRP-conjugated secondary antibodies (BL003A, Biosharp) for 1 hour at room temperature, membranes were visualized with chemiluminescence (ECL, Yeasen).

### Puromycin labeling

The cells at 70%–80% confluence were treated with puromycin (final concentration: 10 μg/mL) for 10 min before being split, and then lysed on ice in SDS-PAGE protein sample loading buffer (P0286, Beyotime). The proteins were transferred to a 0.2 μm Immobilon-PSQ (ISEQ00010, Merck Millipore). The membranes were blocked for 1 hour in TBS containing 5% nonfat milk and 0.1% Tween 20, followed by incubation with puromycin antibodies (1:5000 dilution) overnight at 4 °C. The membrane was visualized using enhanced chemiluminescence.

### Flow cytometry

The cells were harvested, fixed in 75% ethanol, and stained with a commercial kit (C1052, Beyotime) according to the manufacturer’s instructions, followed by cell cycle analysis using Cytoflex (Beckman Coulter) and data analysis with FlowJo (Tree Star) to determine the percentage of cells in G0/G1, S, and G2/M phases.

### Ribosome profiling

Ribosome profiling was performed following the protocol in our previous study [51], with a few modifications according to the previous study [79]. In brief, RAW264.7 cells were seeded in 6 cm dishes and cultured. After treatment with LPS for the indicated times (0, 6, 12, and 24 hours), the cells were washed with cold PBS. Then, 300 μl of lysis buffer was added, and the cells were quickly scraped and collected in RNase-free tubes. The sample was centrifuged at 12,000 rpm for 10 min at 4 °C, and the supernatant was sent to Novogene for RNA-seq library construction. For Ribo-seq, approximately 200 μl of the supernatant was treated with RNase I (AM2295, Thermo Fisher Scientific) for 3 hours at 4 °C, followed by RNA extraction using TRIzol LS. The extracted RNA was denatured at 65 °C for 20 min and run on a 15% TBE-Urea gel. The band around 25–35 nt was excised and eluted overnight in RNA elution buffer (300mM NaAc,1 mM EDTA) at 4 °C. The RNA was precipitated with an equal volume of isopropanol. The RNA was treated by T4 Polynucleotide Kinase (M0201L, New England Biolabs), and then polyadenylated using *Escherichia coli* poly(A) polymerase (M0276L, New England Biolabs) at 37 °C for 30 min. The cDNA library was generated using Template switching RT enzyme mix (M0466L, New England Biolabs) according to the manufacturer’s instructions. The cDNA library was amplified by PCR using Phanta flash super-fidelity DNA polymerase (P521-d1, Vazyme) with barcoded primers following the instructions of Illumina sequencing libraries. The PCR product was run on an 8% TBE gel, and a band around 185 bp was excised and recovered using DNA PAGE Gel Extraction Kit (D0058S, Beyotime). The recovered DNA was sent to Novogene for sequencing.

### Sequencing read alignment

The 3′ adaptors of the raw sequencing reads were trimmed using the software Cutadapt [80]. The trimmed reads with length shorter than 15 nt were discarded. The trimmed reads were then aligned to mouse transcriptome using the software STAR [81]. To compile the mouse reference transcriptome, the genome and annotation files were downloaded from EBSEMBL database (GRCm39.107). The protein coding mRNAs were extracted according to the annotation file using a house-hold script. For each gene, the transcript isoform with the longest CDS was selected as a representative transcript for the gene. To avoid ambiguity, reads aligned to multiple positions or with >2 mismatches were excluded.

### Prediction of the ribosome P-site

Constructing Ribo-seq library using a template-switch method leads to a variable 5′ end, due to the ambiguous number of untemplated nucleotides during reverse transcription [82]. We therefore employed a random forest method (“randomForest” package in R), as used in the previous study [83], to predict the position of P-site. As a result, a clear 3-nucleotide periodicity of Ribo-seq reads was observed, and the overall in-frame rate reached approximately 74%, indicating high Ribo-seq quality and accurate P-site assignment (S1 and S2 Figs).

### Differential analysis of gene expression

We counted the number of Ribo-seq reads or RNA-seq reads aligned to the CDS of individual mRNAs using a house-hold script. The differential analysis for Ribo-seq or RNA-seq between samples (e.g., LPS 0 hour versus LPS 6 hours) was done by the software DESeq2 [84]. mRNAs with total reads in two samples <10 were excluded from analysis. mRNAs with false discovery rate (FDR) < 0.05 and fold change >1 were defined as significantly upregulated mRNAs. mRNAs with FDR < 0.05 and fold change <1 were significantly downregulated mRNAs. The significantly changed mRNAs were then analyzed by DAVID [85] to identify enriched biological processes.

### Analysis of variance (ANOVA)

To identify gene clusters with distinct expression patterns at different time points after LPS treatment, we performed ANOVA using the R function “aov”. The resulting *P* values were adjusted for multiple comparisons using the FDR method. mRNAs with FDR < 0.05 were considered significantly differentially expressed. These mRNAs were subsequently clustered and visualized using the “heatmap.2” function in R, with “ward.D” as the hierarchical clustering method. Based on the clustering results, the differentially expressed mRNAs were divided into six groups using the “cutree” function in R. Each group was then analyzed using DAVID to identify enriched biological processes.

To visualize the expression patterns of individual mRNAs in heatmaps, we applied z-score normalization across time points for each gene using the following equation:


$${z-score}_{ij}=\frac{{value}_{ij}-\mu_{i}}{\sigma_{i}}\;$$ (1)

where ${value}_{ij}$ refers to the expression level (e.g., RPKM or TE) of gene i at time point $j\times u_{i}$ and $\sigma_{i}$ refer to the mean and standard deviation, respectively, of the expression values of gene i across all time points.

### Analysis of codon usage bias

We used the RSCU index to measure the codon usage bias [45]. The RSCU is calculated using following equation.


$${RSCU}_{ij}=\frac{x_{ij}}{\frac{\text{1}}{n_{i}}\sum_{j=\text{1}}^{n_{j}}x_{ij}}$$ (2)

where $x_{ij}$ refers to the frequency of codon j encoding amino acid i in a specific gene cluster. $n_{i}$ refers to the total number of codons that encode the amino acid i.

### Calculation of codon occupancy at ribosomal A-site

For each mRNA, ribosome footprints mapped to the same codons within the coding sequence (CDS) were counted and normalized by the average footprint density across the CDS. Only codons positioned at the ribosomal A-site were considered. mRNAs with total CDS footprints <32 were excluded. Footprints with A-site located to the first and last 30 codons were also excluded. The normalized ribosome occupancies at the same codons were averaged over the transcriptome.

### Identifying ribosomal pausing sites

We first counted the number of footprints on individual mRNA codons. mRNAs with total read counts <10 were excluded from analysis. Then, we applied a zero-truncated binomial negative model to determine the codons with a statistically significant (*P* < 0.05) number of read counts [86,87].

### Identifying uORFs

We applied our previously established method to identify translated uORF based on Ribo-seq data in macrophages [51]. In brief, all potential uORFs were extracted, defined as regions starting with AUG, CUG, UUG, or GUG, and ending with UAG, UGA, or UAA. We then performed a Wilcoxon rank-sum test to determine whether there were significantly more in-frame reads compared to the other two frames, a similar method developed in the previous study [88]. The resulting two *P* values were combined using a Stouffer’s method. The *P* values were then corrected using FDR method. The uORFs with FDR < 0.1 were considered to be robustly translated.

### mRNA stability analysis

The cells at 70% to 80% confluence were treated with LPS at the indicated times, followed by Actinomycin D (ActD, 5 μg/ml) treatment with 0, 1, or 3 hours. Total RNA was extracted following the methods mentioned above. To assess transcriptome stability, total RNA was sent to Novogene for RNA-seq library construction and sequencing. Data analysis was performed using the same pipeline as described above for Ribo-seq. mRNA stability was calculated using following equations.


$$\text{ln}\left(\frac{{RPKM}_{t}}{R{PKM}_{t\text{0}}}\right)=-\lambda t$$ (3)

$$t_{\text{1}/\text{2}}=\frac{\text{ln}(\text{2})}{\lambda }$$ (4)

where ${RPKM}_{t}$ and ${RPKM}_{t\text{0}}$ refer to the RPKM values at time points t and 0 hours after ActD treatment, respectively. The slope λ was estimated by a linear regression. Only the sequences with estimated *P* value < 0.05 and *R*^2^ > 0.5 were used in downstream analysis.

To estimate the stability of individual mRNAs using qPCR analysis, relative mRNA level of the indicated mRNA (e.g., *Cdk2*, *Cdk4*, and *Cdk6*) was calculated based on qPCR analysis. mRNA stability is calculated using eqs. (3) and (4).

### Calculation of codon stabilization coefficient (CSC)

We calculated CSC values based on method in previous studies. In brief, a CSC value refers to the Pearson correlation between the frequency of a codon in an mRNA and the stability of the mRNA. A positive value for a codon indicates that the codon stabilizes the mRNA, whereas a negative value indicates that the codon destabilizes the mRNA.

## Supporting information

S1 Fig Ribo-seq analysis of macrophages in response to LPS treatment. **(A)** Heatmap showing the correlation between RNA-seq and Ribo-seq samples treated with LPS for 0, 6, 12, or 24 hours (*n* = 2 biological replicates). **(B)** RT-qPCR results of classical macrophage inflammatory genes at indicated time (0, 6, 12, and 24 hours) under LPS treatment. Error bars represent mean ± SEM, unpaired two tailed *t* test, *** *P* < 0.001, *n* = 6 biological replicates. **(C)** Length of footprint reads. A typical footprint length distribution of Ribo-seq, with a median length around 29 nt, is observed across all samples (*n* = 2 biological replicates). **(D)** Regions (5′ UTR, CDS, and 3′ UTR) of footprint reads are located to. Majority of reads (~85%) were mapped to CDS (*n* = 2 biological replicates). The data underlying the graphs shown in the figure can be found in S1 Data.
(PDF)

S2 Fig Quality analysis of Ribo-seq. **(A)** Aggregation plotting showing mean Ribo-seq reads around the start and stop codon. A typical 3-nt periodicity can be observed in all samples (*n* = 2 biological replicates). **(B)** Fraction of Ribo-seq reads in different reading frames. “0” indicates in-frame reads, “1” and “2” indicate the frame 1 and frame 2 reads, respectively. The average in-frame rate for all samples is around 72%, suggesting a high-quality of Ribo-seq dataset (*n* = 2 biological replicates). The data underlying the graphs shown in the figure can be found in S1 Data.
(PDF)

S3 Fig Analysis of inflammatory and translational response to LPS in macrophages. **(A)** Principal component analysis (PCA) plot of RNA-seq and Ribo-seq samples. mRNAs with RPKM > 1 were used for analysis (*n* = 2 biological replicates). **(B)** Scatter plots illustrating expression changes at the level of transcription (X-axis, RNA-Seq) and translation (Y-Axis, Ribo-Seq). mRNAs with fold change of TE > 2 were defined as the group “TE up”, and mRNAs with fold change of TE < 0.5 were defined as the group “TE down”. A mean value of the two biological replicates was used. **(C)** Box plots showing the distribution of gene expression levels in 6 clusters (based on the data in Fig 1D) at both translational and transcriptional levels. A mean value of the two biological replicates was used. **(D)** Heatmap showing the translation efficiency (TE) values of ribosomal proteins and initiation factors. TE was calculated as the ratio of Ribo-seq over RNA-seq. A mean value of the two biological replicates was used. **(E)** Quantification of the immunoblot of key proteins in mTOR pathway, related to the western blot of Fig 1G (*n* = 3 biological replicates). The data underlying the graphs shown in the figure can be found in S1 Data.
(PDF)

S4 Fig Analysis of translational response to LPS in mouse BMDMs. **(A)** A representative western blot of mouse BMDMs before and after LPS treatment at indicated timepoints. **(B)** Quantification of the immunoblot of key proteins in mTOR pathway, related to the western blot of S4A Fig (*n* = 3 biological replicates). **(C)** Puromycin labeling assay in BMDMs before and after LPS treatment. Bar plots show the relative levels of puromycin-labeled nascent chains quantified by densitometry. Error bars represent mean ± SEM, unpaired two tailed *t* test, *** *P* < 0.001, *n* = 3 biological replicates. The data underlying the graphs shown in the figure can be found in S1 Data. Raw blot images can be found in S1 Raw Images.
(PDF)

S5 Fig Analysis of cell cycle-related mRNAs during macrophage polarization. **(A)** RT-qPCR analysis for checkpoints associated with different phases of the cell cycle at different timepoints. Error bars represent mean ± SEM, unpaired two tailed *t* test, **P < 0.01, *** *P* < 0.001, *n* = 3 biological replicates. **(B)** Original RPKM values of cell cycle-related mRNAs detected by Ribo-seq and RNA-seq. Translation efficiency (TE) was also calculated by the ratio of Ribo-seq over RNA-seq. **(C)** Flow cytometry analysis of cell cycle. Error bars represent mean ± SEM, unpaired two tailed *t* test, **P < 0.01, *** *P* < 0.001, *n* = 3 biological replicates. **(D)** The volcano plots (left) depicting changes in protein translation in mouse BMDM cells treated with LPS for 6 or 24 hours, compared to the control group. The bar plots (right) display the results of GO analysis for upregulated and downregulated genes. The data underlying the graphs shown in the figure can be found in S1 Data.
(PDF)

S6 Fig 5′ UTR features of cell cycle-related mRNAs. **(A–C)** Box plot showing length **(A)**, GC content **(B)**, and folding free energy **(C)** of 5′ UTR in cluster 4 (cell cycle-related mRNAs) compared to other mRNAs. Wilcoxon tests were performed between each pair of samples. **(D)** The scatter plot (left) shows initiation potential values for individual transcripts in 0 hours and 6 hours of LPS treatment. Transcripts with a fold change in initiation potential >2 are highlighted in red (high), and those with a fold change <0.5 are highlighted in blue (low). The bar plot (right) displays enriched biological processes among mRNAs with high initiation potential in 6 hours of post-LPS treatment. The data underlying the graphs shown in the figure can be found in S1 Data.
(PDF)

S7 Fig Codon usage analysis in macrophages response to LPS treatment. **(A)** Mouse BMDMs with or without LPS treatment (24 hour) were subjected to proteomics analysis. The mRNAs with increased or decreased protein levels were extracted, and the codon usage in the two groups of mRNAs was calculated. Fold change of y-axis indicates the fold change of codon usage in mRNA group with increased protein level over codon usage in mRNA group with decreased protein level. Wilcoxon tests were performed between each pair of samples. **(B)** RT-qPCR results of reporter mRNAs at indicated time (0, 6, 12, and 24 hours) under LPS treatment. Error bars represent mean ± SEM, *n* = 3 biological replicates. **(C)** RNA stability analysis between cells with or without LPS treatment. Line plots showing relative mRNA levels after ActD treatment. Half-life values (*t*_*1/2*_) for each LPS treated samples were estimated using a linear regression (see Methods in the main text). unpaired two tailed *t* test was performed to determine the statistical significance between two samples (*n* = 3, the values in parentheses represent the half-life values ± SD). The data underlying the graphs shown in the figure can be found in S1 Data.
(PDF)

S8 Fig Codon usage bias and mRNA stability in mouse cell lines. **(A, B)** show the correlation between codon frequency and mRNA stability from mouse ESC or EB cells. Wilcoxon tests were performed between each pair of samples. **(C)** RNA stability analysis between cells with or without LPS treatment. Line plots showing relative mRNA levels after ActD treatment. Half-life values (*t*_*1/2*_) for each LPS treated samples were estimated using a linear regression (see Methods in the main text). Unpaired two tailed *t* test was performed to determine the statistical significance between two samples (*n* = 3, the values in parentheses represent the half-life values ± SD). The line plots in bottom panels showing mRNA levels after DMSO treated at different points. DMSO treatment did not lead to increased mRNA levels in LPS-treated cells, arguing against the enhanced mRNA stability observed following LPS stimulation is not due to DMSO treatment. The data underlying the graphs shown in the figure can be found in S1 Data.
(PDF)

S9 Fig Expression of tRNAs measured by small RNA-seq. All tRNAs were grouped into AU3 or GC3 codons based on their decoding codons.
(PDF)

S1 Table uORFs identified in this study. (XLSX)

S2 Table Primers used in this study. (XLSX)

S1 Data Numerical values underlying all graphs in the main body and Supporting information. (XLSX)

S1 Raw Images Uncropped version of all western blot images and flow gating in the main body and Supporting information.
(PDF)

## Abbreviations

ANOVA: analysis of variance
BMDMs: bone marrow-derived macrophages
CDS: coding sequence
CSC: codon stabilization coefficient
FDR: false discovery rate
Il-1β: interleukin-1 beta
Il-6: interleukin-6
JNK: C-Jun N-terminal kinase
LPS: lipopolysaccharide
mTOR: mammalian target of rapamycin
mTORC1: mTOR complex 1
PCA: principal component analysis
RSCU: relative synonymous codon usage
TE: translation efficiency;
Tnf-α: tumor necrosis factor-alpha
TOP: terminal oligopyrimidine tract
uORFs: upstream open reading frames
