# Supplementary figures and images for: Unconventional codon usage bias mediates mRNA translational dynamics in macrophages
Source: PLoS Biol. 2025 Sep 18;23(9):e3003403. doi: 10.1371/journal.pbio.3003403 (PMC12456811; doi:10.1371/journal.pbio.3003403)

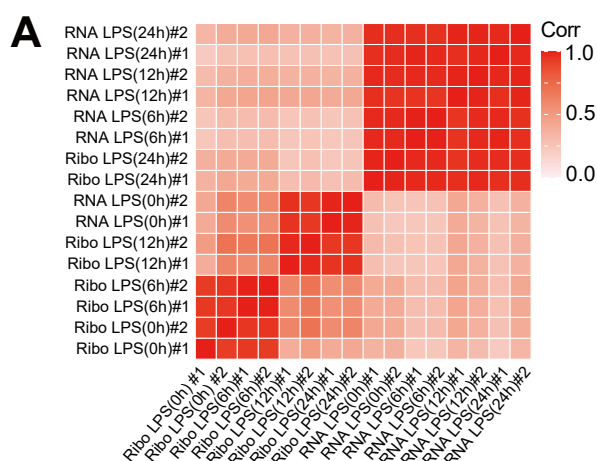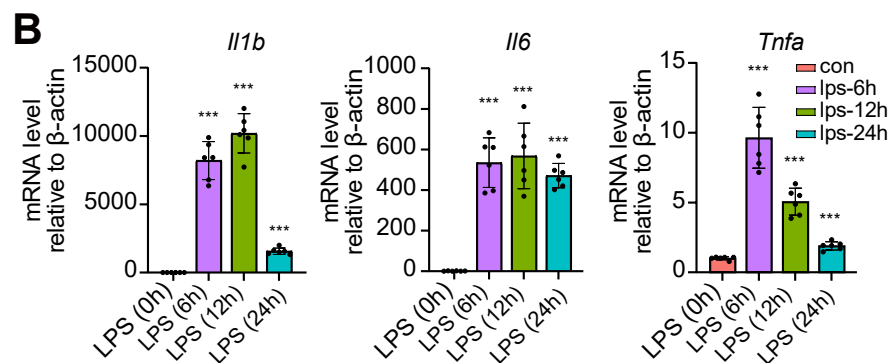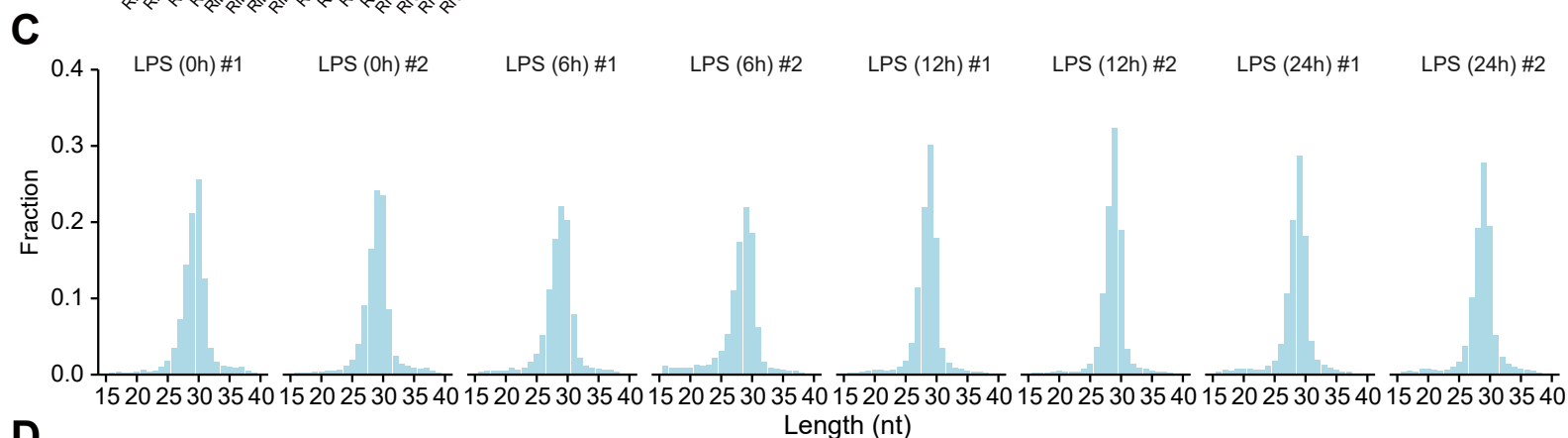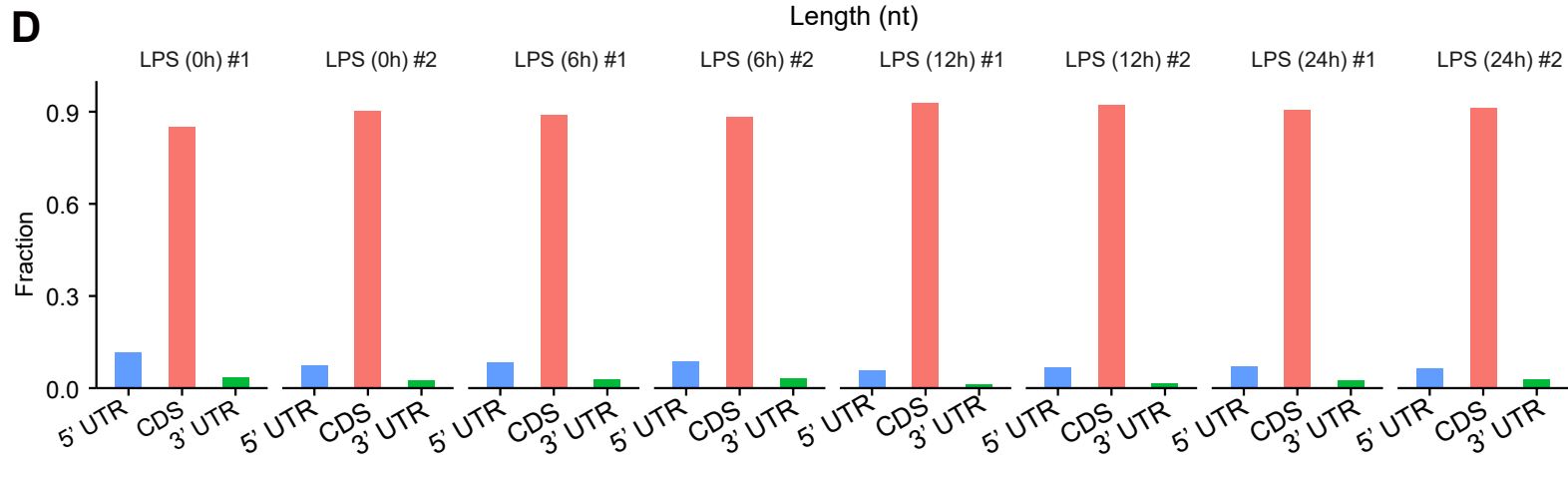

Supplement: S1 Fig — (A) Heatmap showing the correlation between RNA-seq and Ribo-seq samples treated with LPS for 0, 6, 12, or 24 hours (n = 2 biological replicates). (B) RT-qPCR results of classical macrophage inflammatory genes at indicated time (0, 6, 12, and 24 hours) under LPS treatment. Error bars represent mean ± SEM, unpaired two tailed t test, *** P < 0.001, n = 6 biological replicates. (C) Length of footprint reads. A typical footprint length distribution of Ribo-seq, with a median length around 29 nt, is observed across all samples (n = 2 biological replicates). (D) Regions (5′ UTR, CDS, and 3′ UTR) of footprint reads are located to. Majority of reads (~85%) were mapped to CDS (n = 2 biological replicates). The data underlying the graphs shown in the figure can be found in S1 Data. (PDF) [file pbio.3003403.s001.pdf]

**A**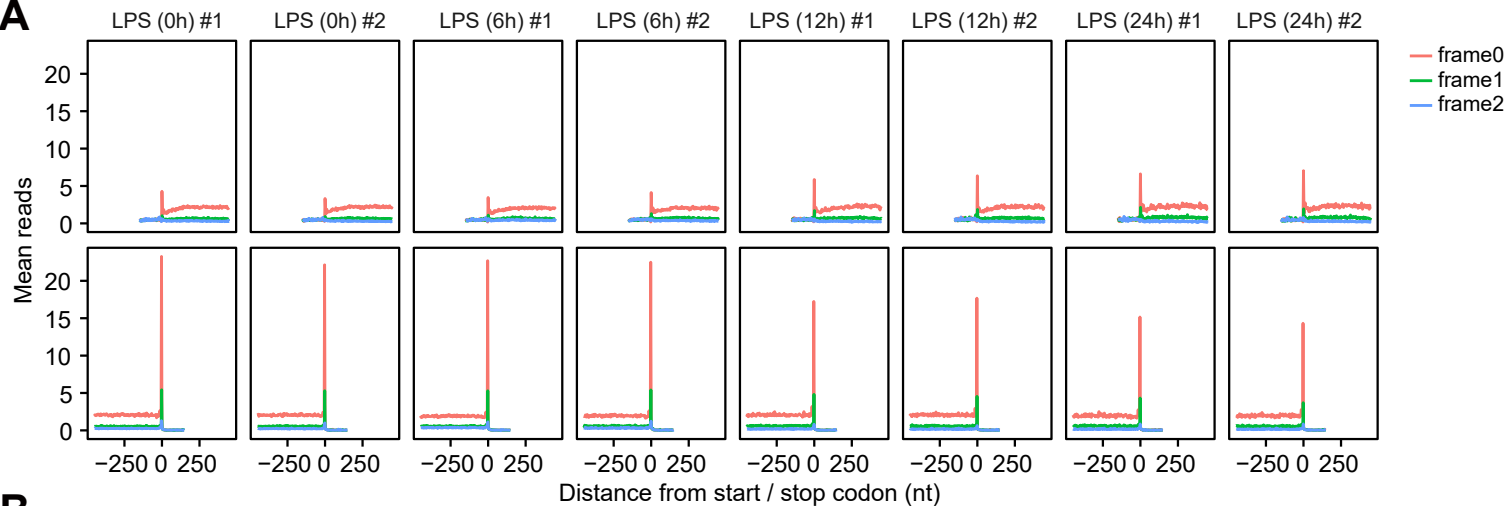**B**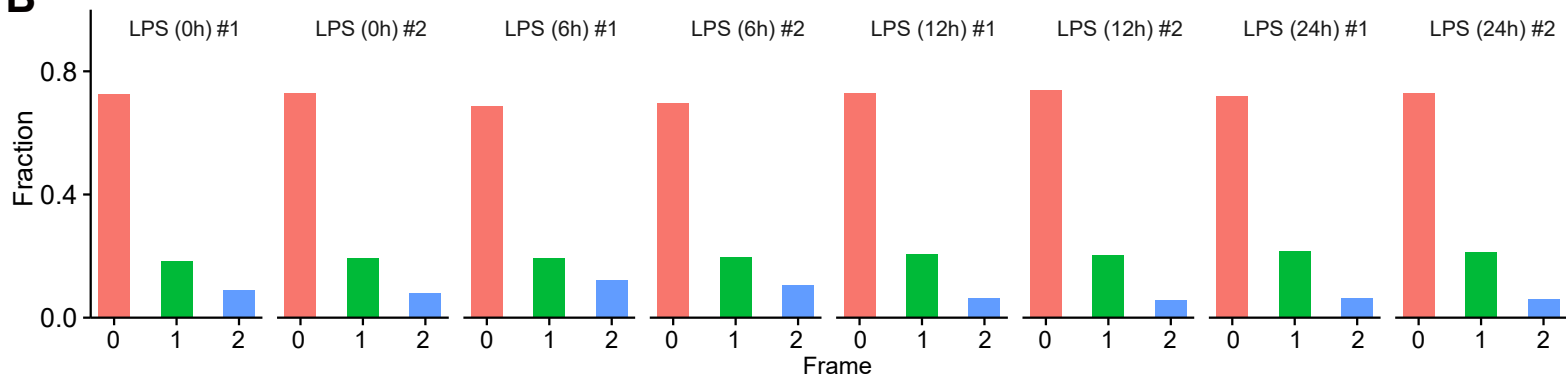

Supplement: S2 Fig — (A) Aggregation plotting showing mean Ribo-seq reads around the start and stop codon. A typical 3-nt periodicity can be observed in all samples (n = 2 biological replicates). (B) Fraction of Ribo-seq reads in different reading frames. “0” indicates in-frame reads, “1” and “2” indicate the frame 1 and frame 2 reads, respectively. The average in-frame rate for all samples is around 72%, suggesting a high-quality of Ribo-seq dataset (n = 2 biological replicates). The data underlying the graphs shown in the figure can be found in S1 Data. (PDF) [file pbio.3003403.s002.pdf]

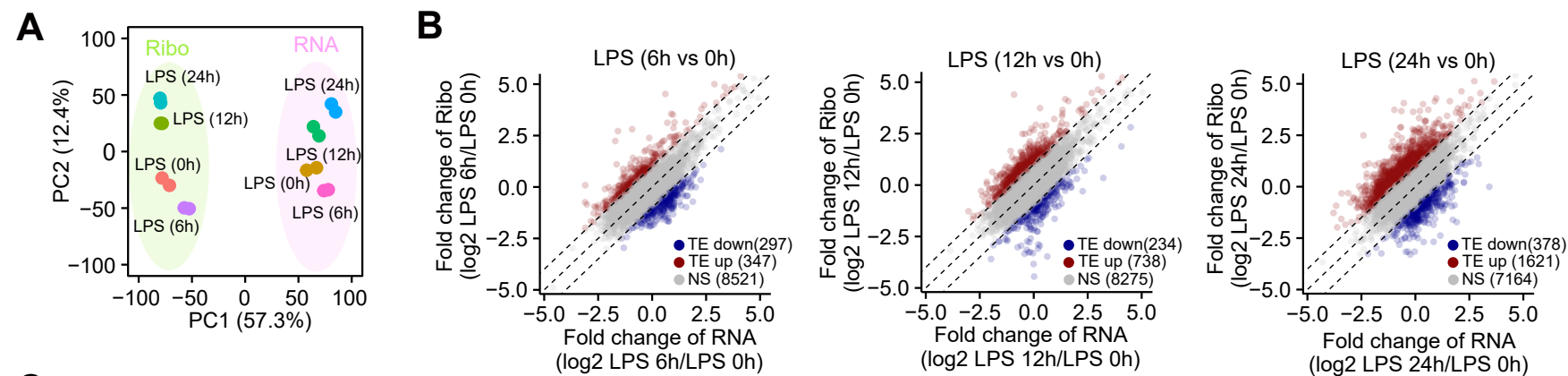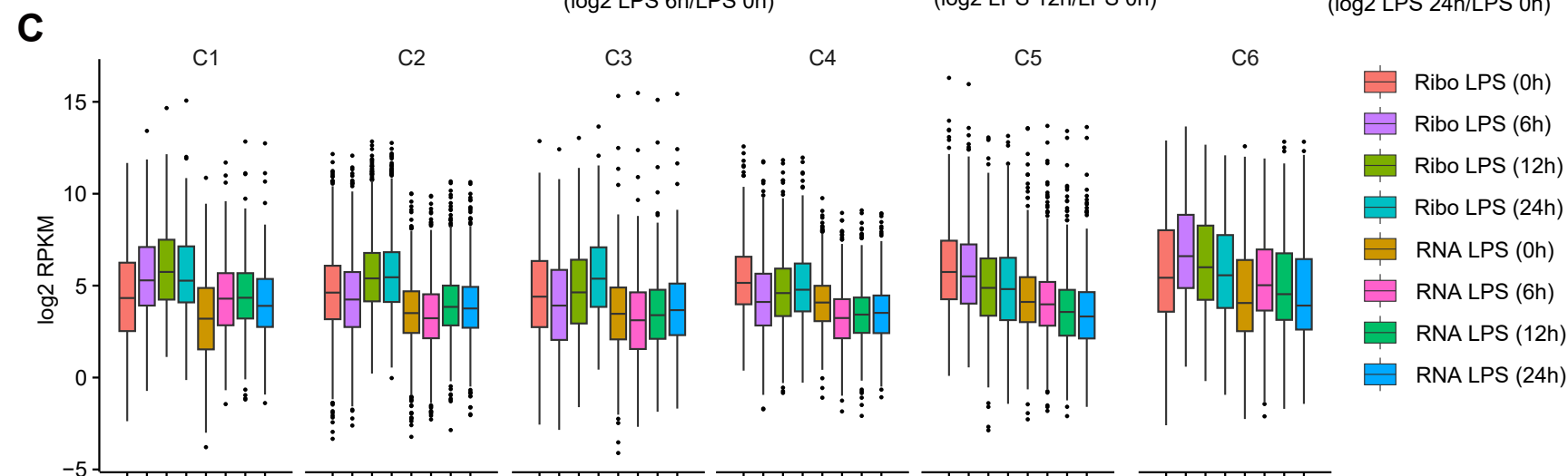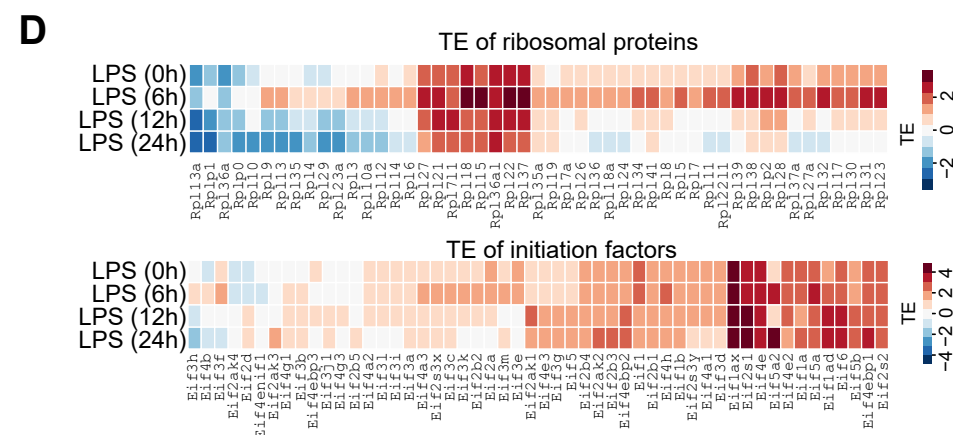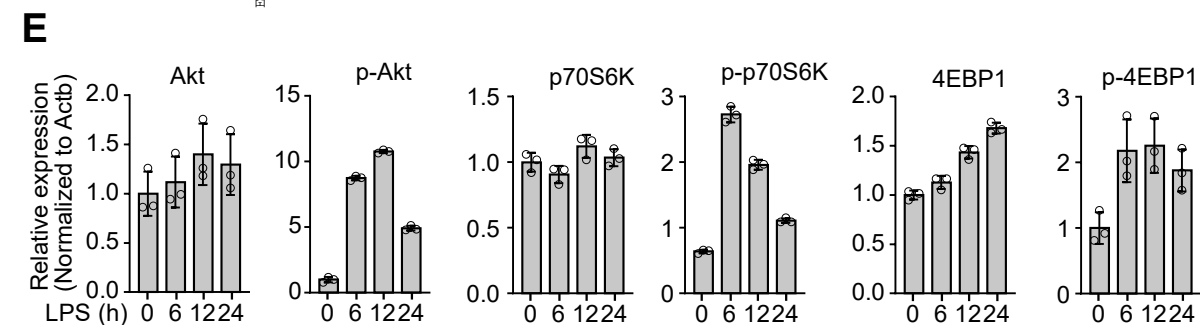

Supplement: S3 Fig — (A) Principal component analysis (PCA) plot of RNA-seq and Ribo-seq samples. mRNAs with RPKM > 1 were used for analysis (n = 2 biological replicates). (B) Scatter plots illustrating expression changes at the level of transcription (X-axis, RNA-Seq) and translation (Y-Axis, Ribo-Seq). mRNAs with fold change of TE > 2 were defined as the group “TE up”, and mRNAs with fold change of TE < 0.5 were defined as the group “TE down”. A mean value of the two biological replicates was used. (C) Box plots showing the distribution of gene expression levels in 6 clusters (based on the data in Fig 1D) at both translational and transcriptional levels. A mean value of the two biological replicates was used. (D) Heatmap showing the translation efficiency (TE) values of ribosomal proteins and initiation factors. TE was calculated as the ratio of Ribo-seq over RNA-seq. A mean value of the two biological replicates was used. (E) Quantification of the immunoblot of key proteins in mTOR pathway, related to the western blot of Fig 1G (n = 3 biological replicates). The data underlying the graphs shown in the figure can be found in S1 Data. (PDF) [file pbio.3003403.s003.pdf]

**A**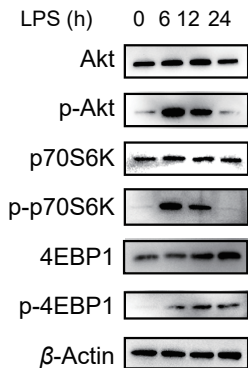**C**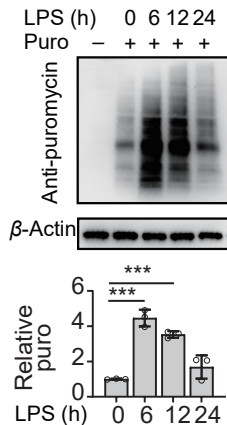**B**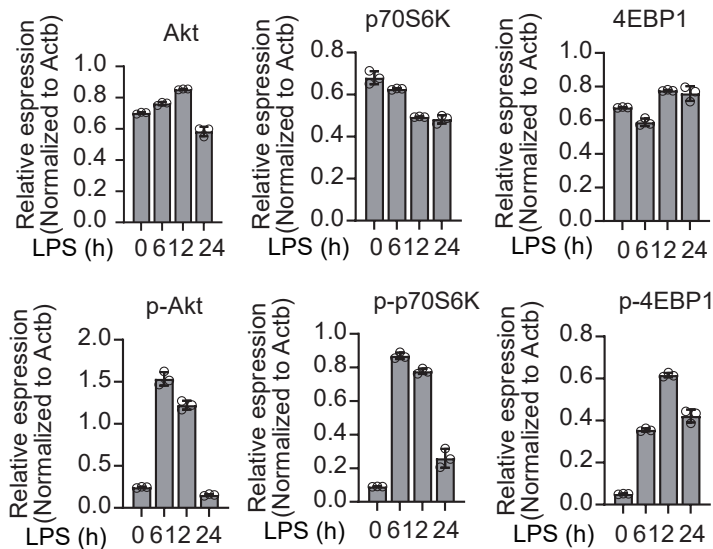

Supplement: S4 Fig — (A) A representative western blot of mouse BMDMs before and after LPS treatment at indicated timepoints. (B) Quantification of the immunoblot of key proteins in mTOR pathway, related to the western blot of S4A Fig (n = 3 biological replicates). (C) Puromycin labeling assay in BMDMs before and after LPS treatment. Bar plots show the relative levels of puromycin-labeled nascent chains quantified by densitometry. Error bars represent mean ± SEM, unpaired two tailed t test, *** P < 0.001, n = 3 biological replicates. The data underlying the graphs shown in the figure can be found in S1 Data. Raw blot images can be found in S1 Raw Images. (PDF) [file pbio.3003403.s004.pdf]

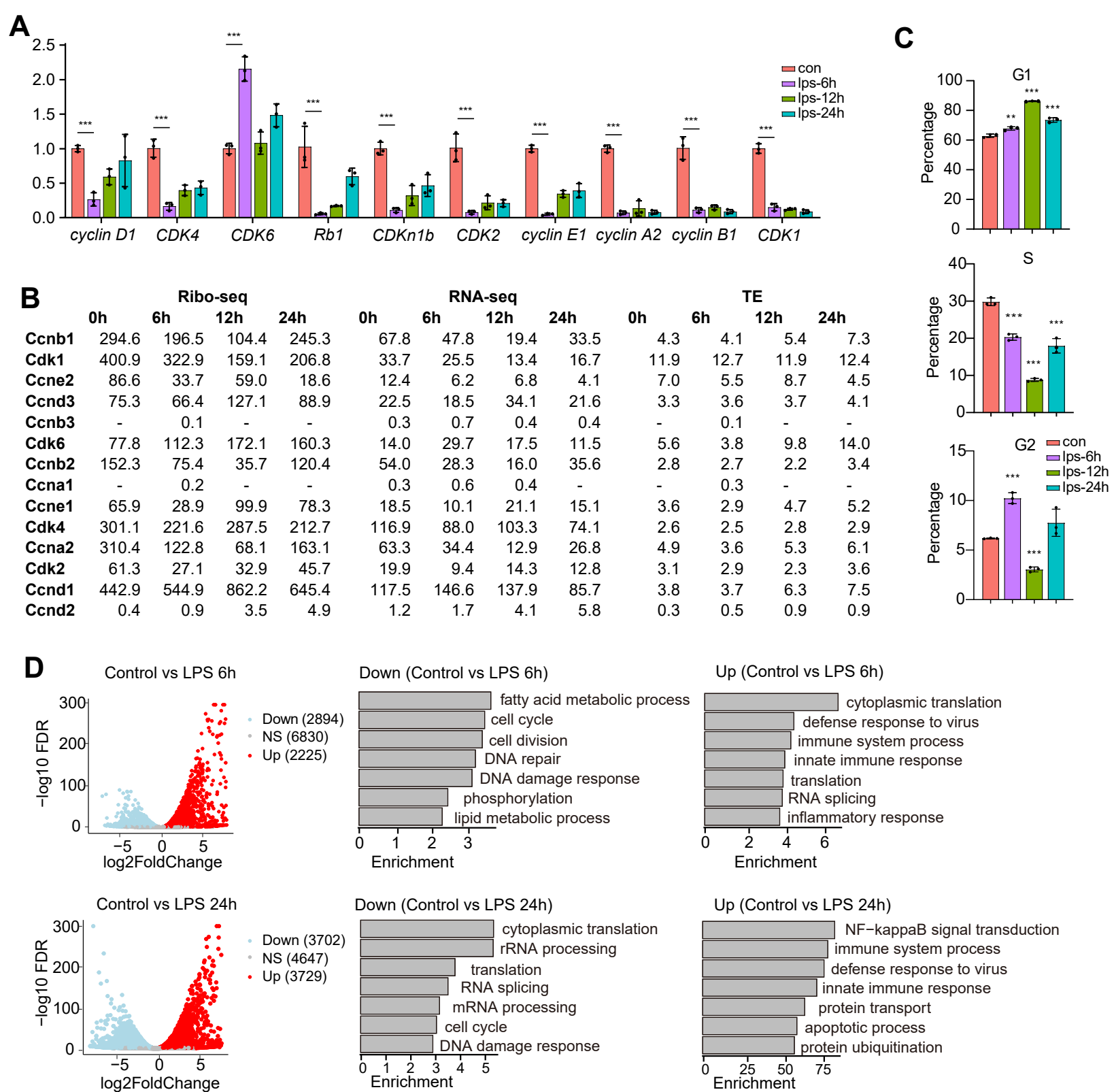

Supplement: S5 Fig — (A) RT-qPCR analysis for checkpoints associated with different phases of the cell cycle at different timepoints. Error bars represent mean ± SEM, unpaired two tailed t test, **P < 0.01, *** P < 0.001, n = 3 biological replicates. (B) Original RPKM values of cell cycle-related mRNAs detected by Ribo-seq and RNA-seq. Translation efficiency (TE) was also calculated by the ratio of Ribo-seq over RNA-seq. (C) Flow cytometry analysis of cell cycle. Error bars represent mean ± SEM, unpaired two tailed t test, **P < 0.01, *** P < 0.001, n = 3 biological replicates. (D) The volcano plots (left) depicting changes in protein translation in mouse BMDM cells treated with LPS for 6 or 24 hours, compared to the control group. The bar plots (right) display the results of GO analysis for upregulated and downregulated genes. The data underlying the graphs shown in the figure can be found in S1 Data. (PDF) [file pbio.3003403.s005.pdf]

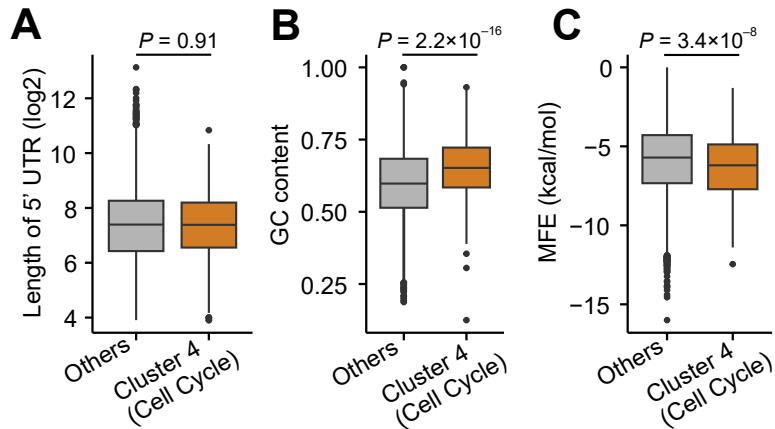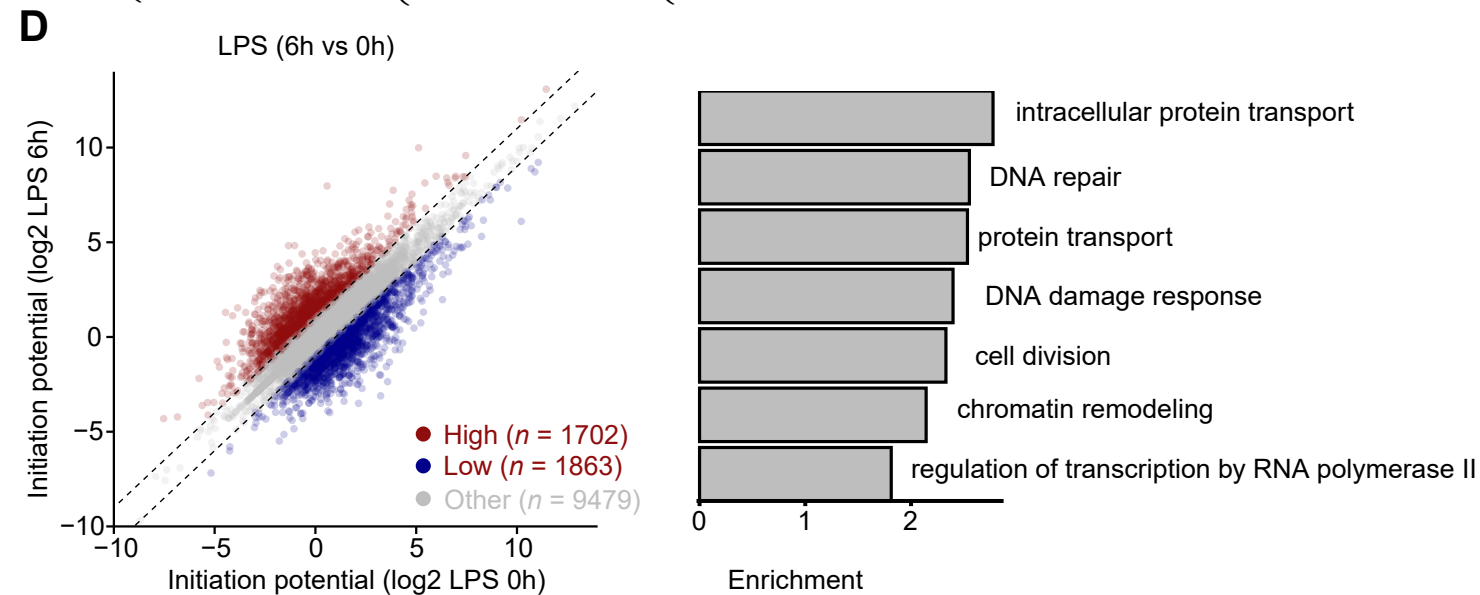

Supplement: S6 Fig — (A–C) Box plot showing length (A), GC content (B), and folding free energy (C) of 5′ UTR in cluster 4 (cell cycle-related mRNAs) compared to other mRNAs. Wilcoxon tests were performed between each pair of samples. (D) The scatter plot (left) shows initiation potential values for individual transcripts in 0 hours and 6 hours of LPS treatment. Transcripts with a fold change in initiation potential >2 are highlighted in red (high), and those with a fold change <0.5 are highlighted in blue (low). The bar plot (right) displays enriched biological processes among mRNAs with high initiation potential in 6 hours of post-LPS treatment. The data underlying the graphs shown in the figure can be found in S1 Data. (PDF) [file pbio.3003403.s006.pdf]

**A**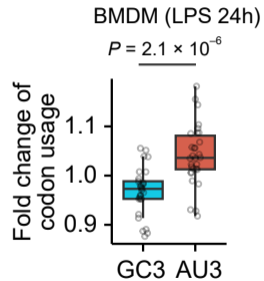**B**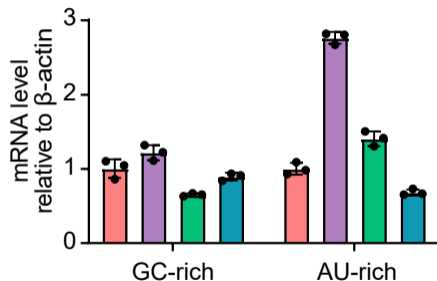**C**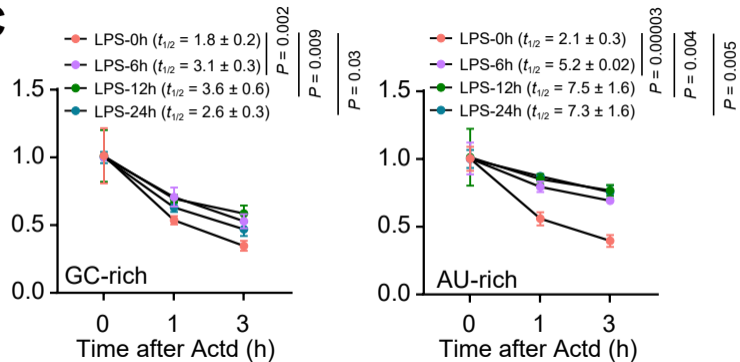

Supplement: S7 Fig — (A) Mouse BMDMs with or without LPS treatment (24 hour) were subjected to proteomics analysis. The mRNAs with increased or decreased protein levels were extracted, and the codon usage in the two groups of mRNAs was calculated. Fold change of y-axis indicates the fold change of codon usage in mRNA group with increased protein level over codon usage in mRNA group with decreased protein level. Wilcoxon tests were performed between each pair of samples. (B) RT-qPCR results of reporter mRNAs at indicated time (0, 6, 12, and 24 hours) under LPS treatment. Error bars represent mean ± SEM, n = 3 biological replicates. (C) RNA stability analysis between cells with or without LPS treatment. Line plots showing relative mRNA levels after ActD treatment. Half-life values (t1/2) for each LPS treated samples were estimated using a linear regression (see Methods in the main text). unpaired two tailed t test was performed to determine the statistical significance between two samples (n = 3, the values in parentheses represent the half-life values ± SD). The data underlying the graphs shown in the figure can be found in S1 Data. (PDF) [file pbio.3003403.s007.pdf]

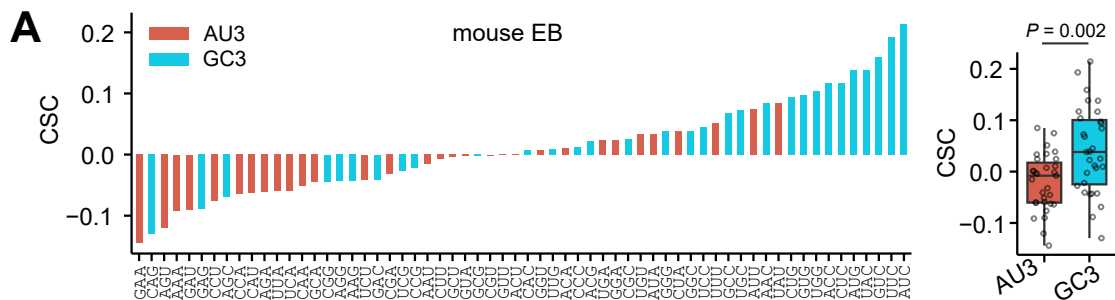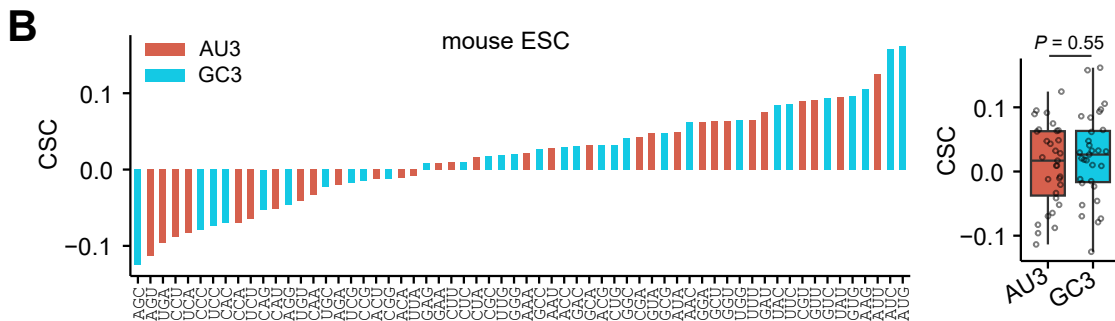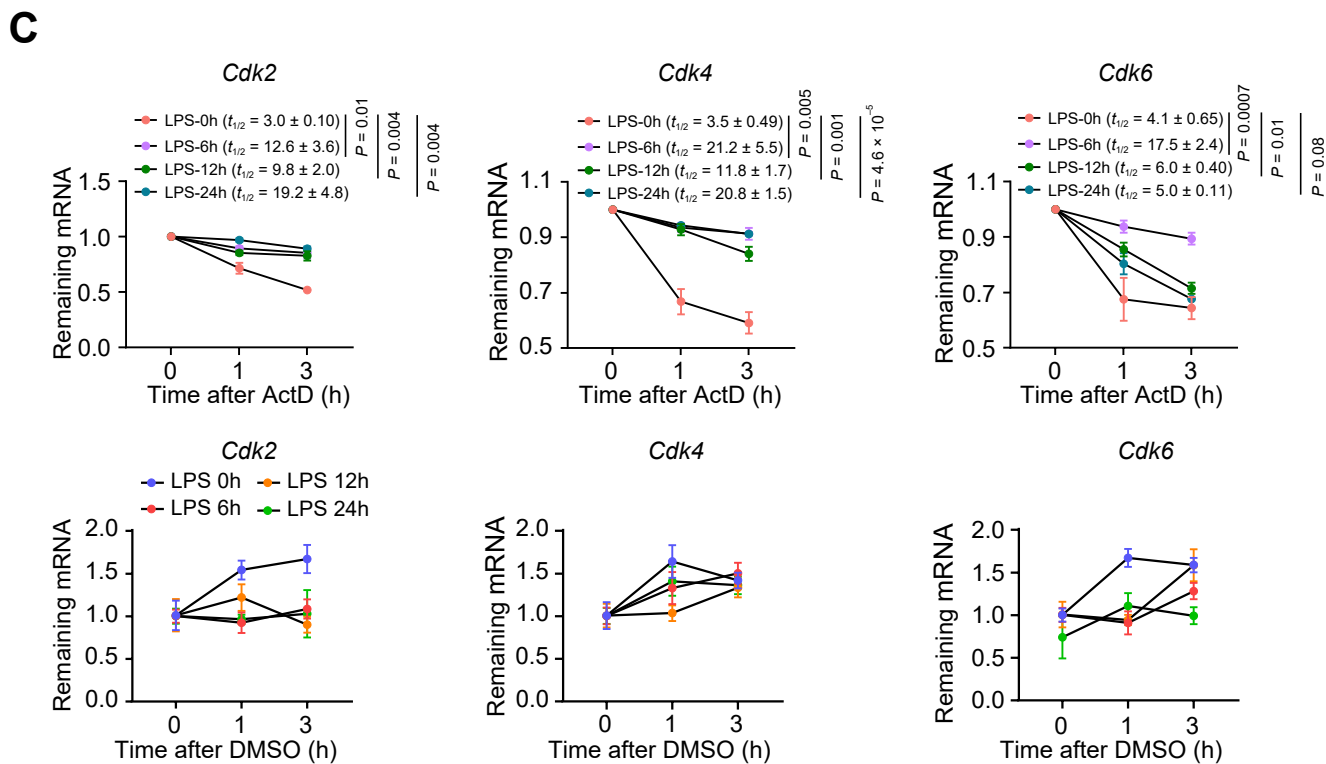

Supplement: S8 Fig — (A, B) show the correlation between codon frequency and mRNA stability from mouse ESC or EB cells. Wilcoxon tests were performed between each pair of samples. (C) RNA stability analysis between cells with or without LPS treatment. Line plots showing relative mRNA levels after ActD treatment. Half-life values (t1/2) for each LPS treated samples were estimated using a linear regression (see Methods in the main text). Unpaired two tailed t test was performed to determine the statistical significance between two samples (n = 3, the values in parentheses represent the half-life values ± SD). The line plots in bottom panels showing mRNA levels after DMSO treated at different points. DMSO treatment did not lead to increased mRNA levels in LPS-treated cells, arguing against the enhanced mRNA stability observed following LPS stimulation is not due to DMSO treatment. The data underlying the graphs shown in the figure can be found in S1 Data. (PDF) [file pbio.3003403.s008.pdf]

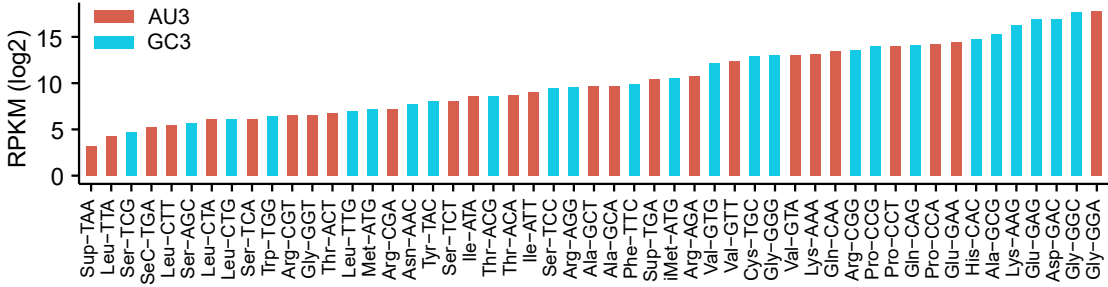

Supplement: S9 Fig — All tRNAs were grouped into AU3 or GC3 codons based on their decoding codons. (PDF) [file pbio.3003403.s009.pdf]
