# Supplementary material for: Unconventional codon usage bias mediates mRNA translational dynamics in macrophages
Source: PLoS Biol. 2025 Sep 18;23(9):e3003403. doi: 10.1371/journal.pbio.3003403 (PMC12456811; doi:10.1371/journal.pbio.3003403)
Supplement: S1 Raw Images — Uncropped version of all western blot images and flow gating in the main body and Supporting information. (PDF) [file pbio.3003403.s013.pdf]

Figure 1G Akt

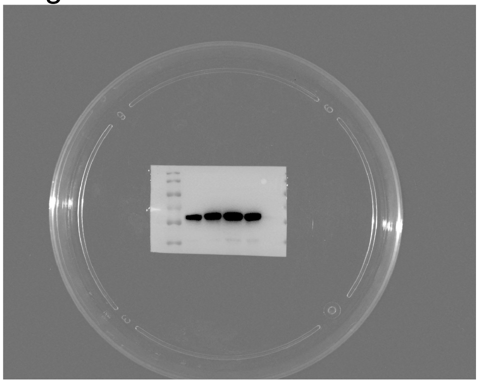

Figure 1G p-Akt

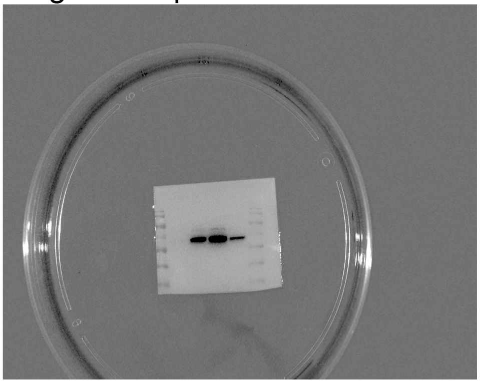

Figure 1G p70S6K

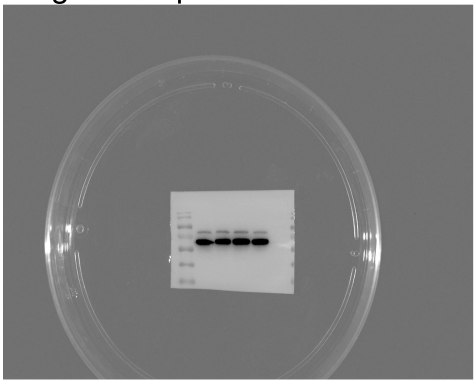

Figure 1G p-p70S6K

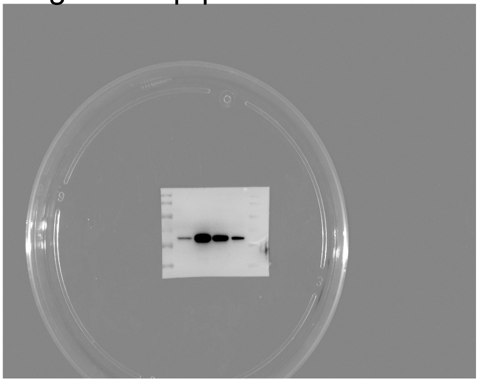

Figure 1G 4EBP1

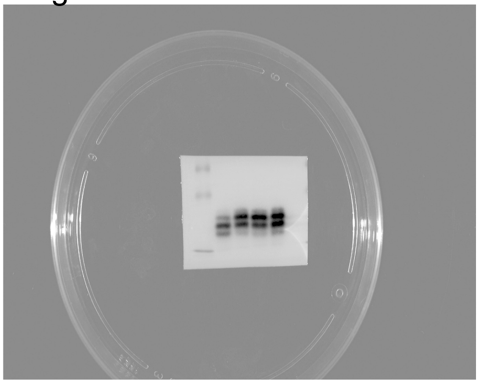

Figure 1G p-4EBP1

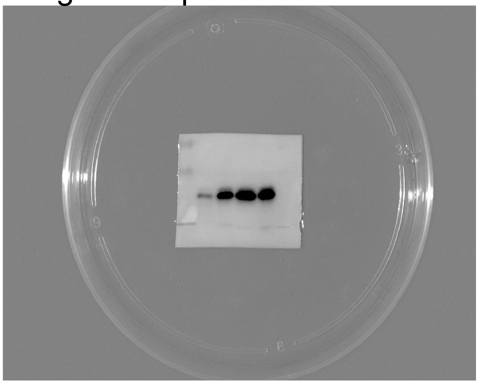

Figure 1G β-actin

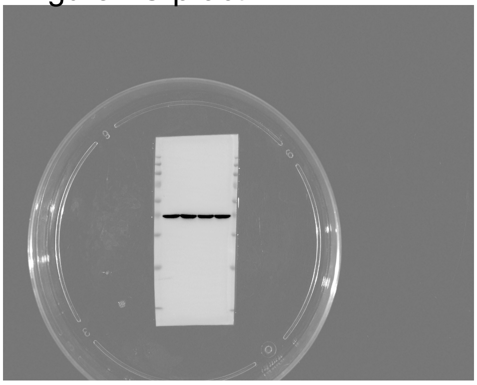

Figure 1H Puro

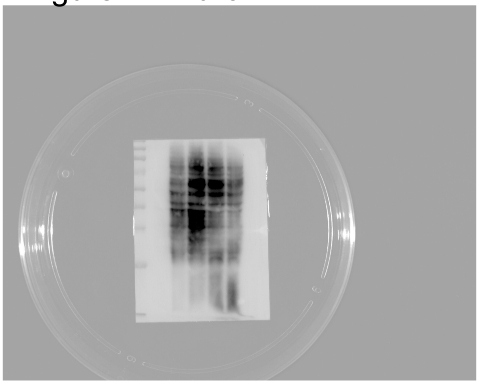

Figure 1H β-actin

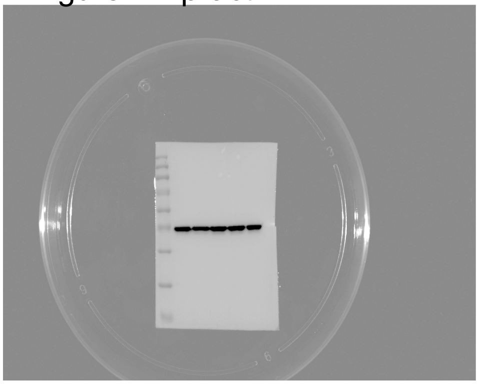

Figure 3E Flag (up)

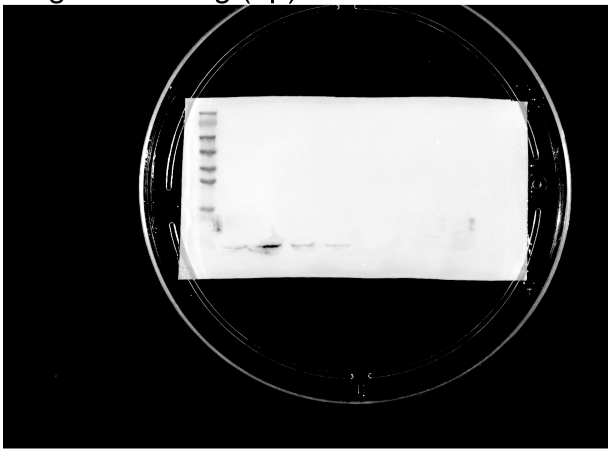

Figure 3E Fluc (down)

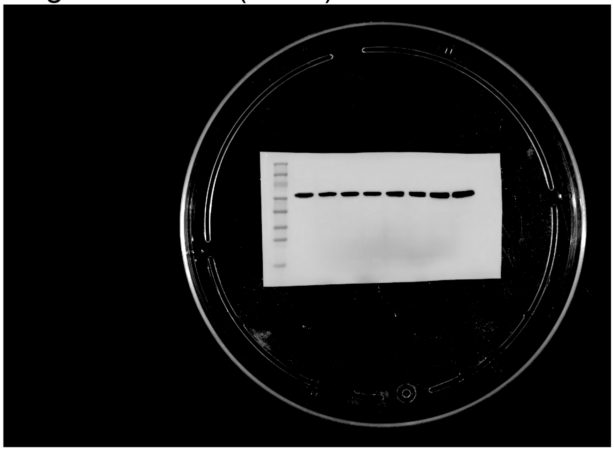

Figure 3E Flag (up)

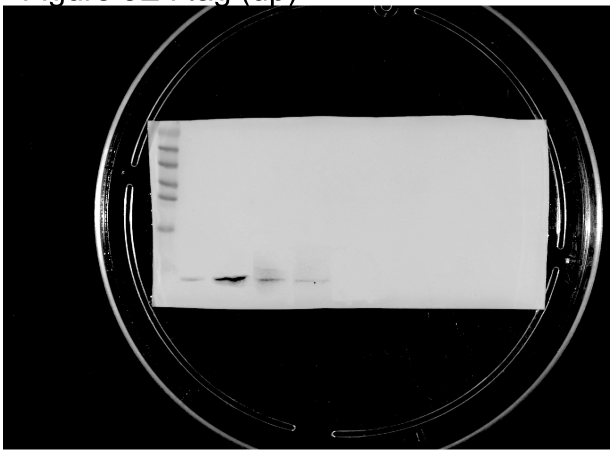

Figure 3E Fluc (down)

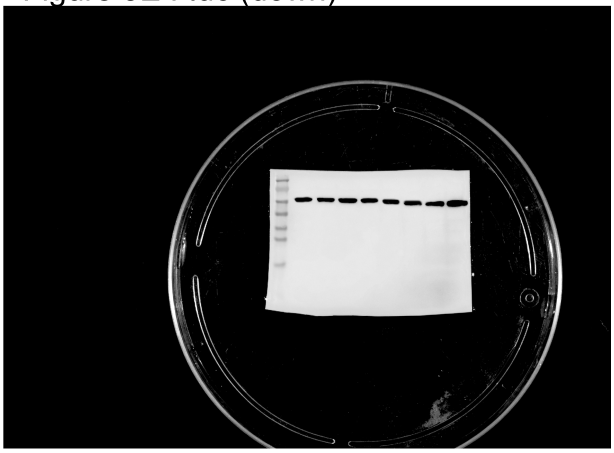

Figure S4A Akt

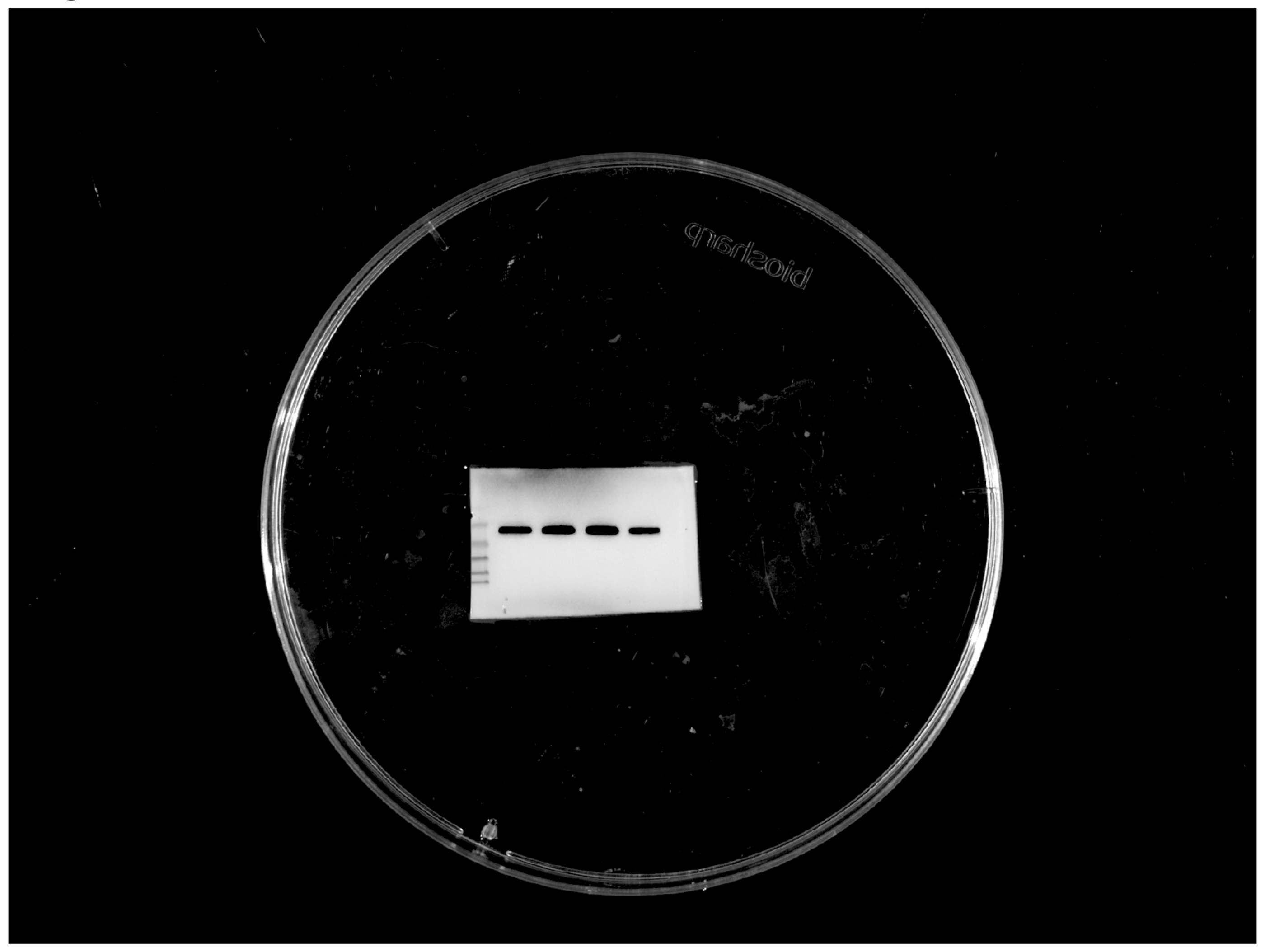

Figure S4A p-Akt

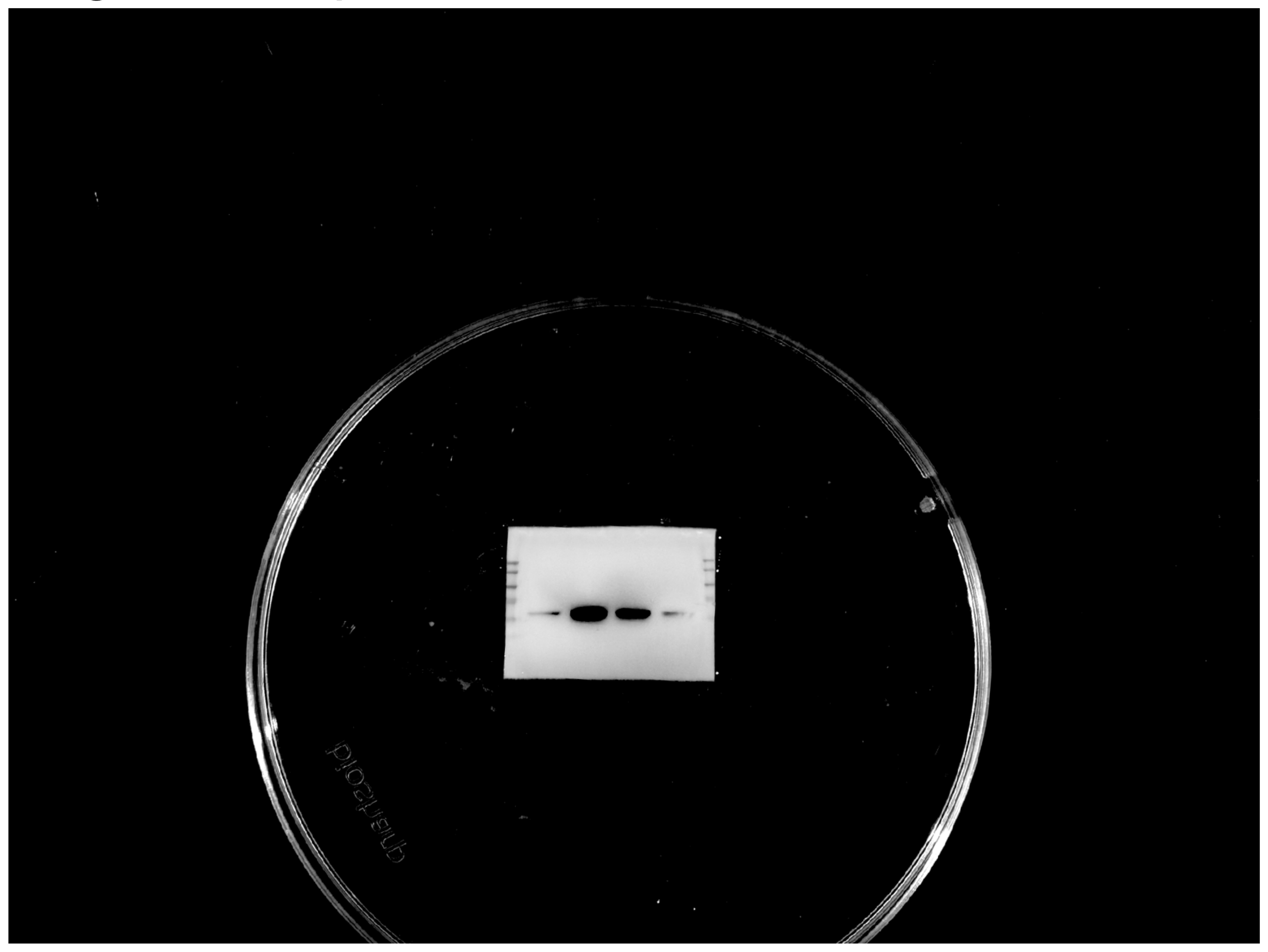

Figure S4A p70S6K

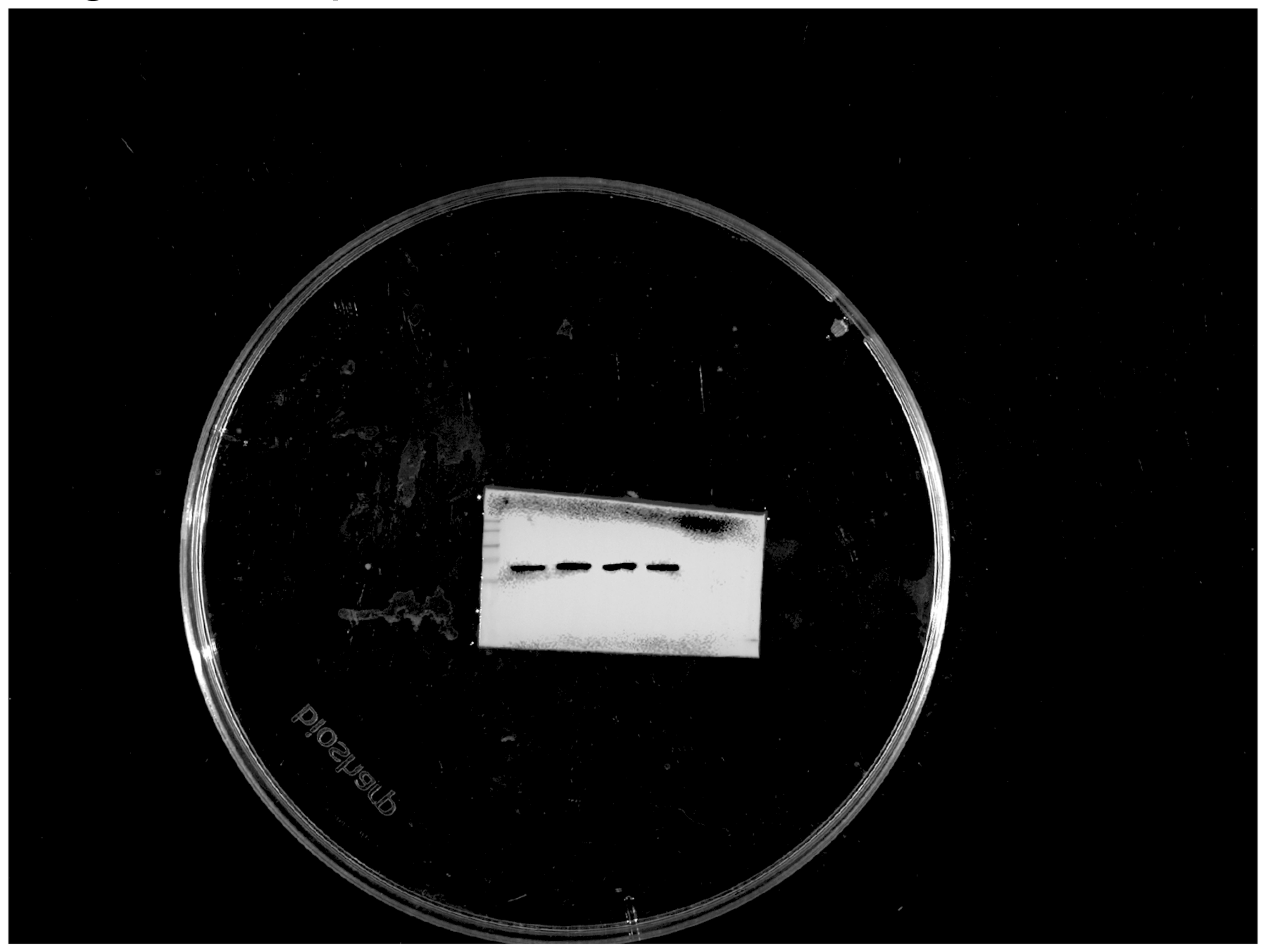

Figure S4A p-P70S6K

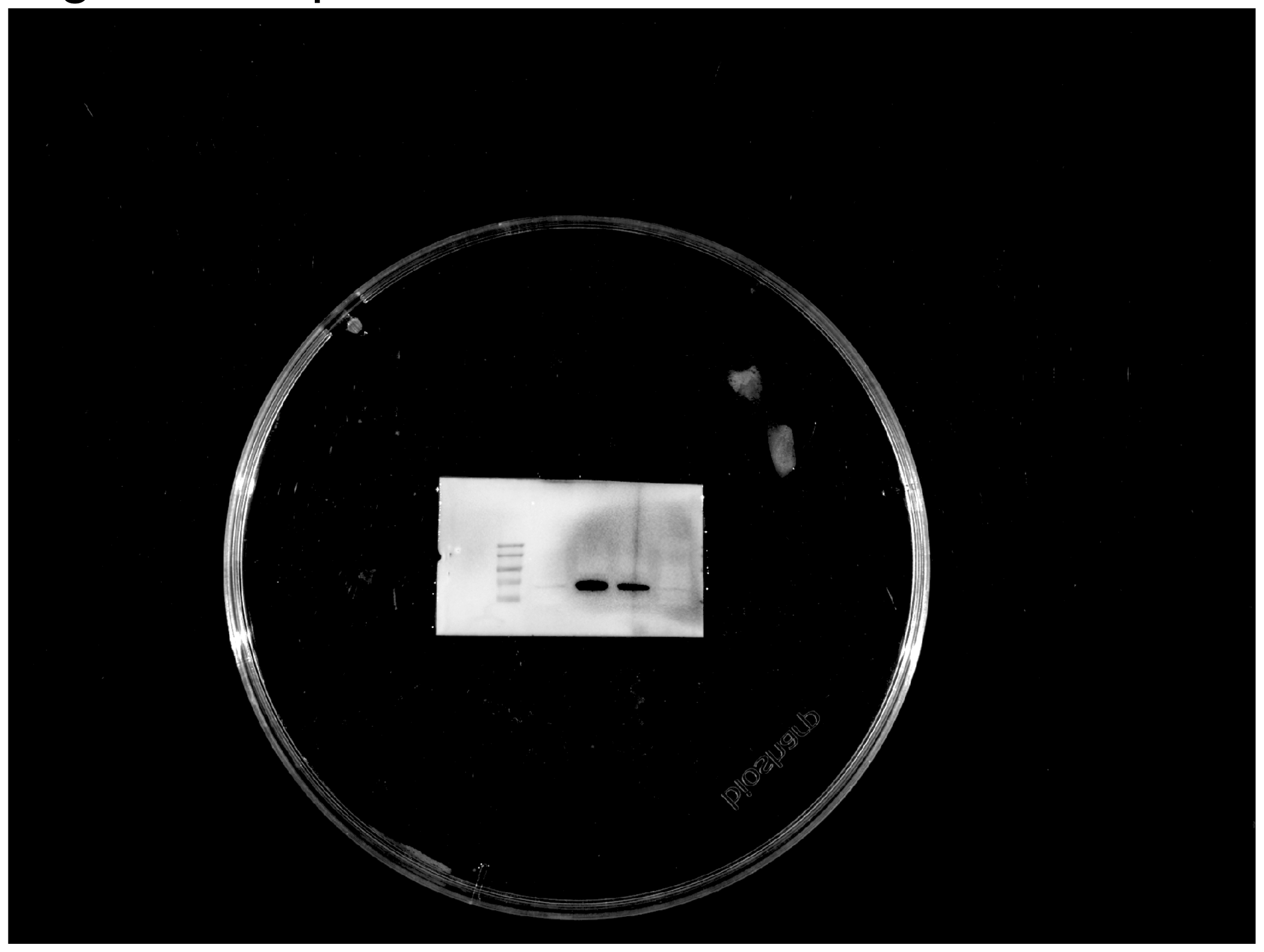

Figure S4A 4EBP1

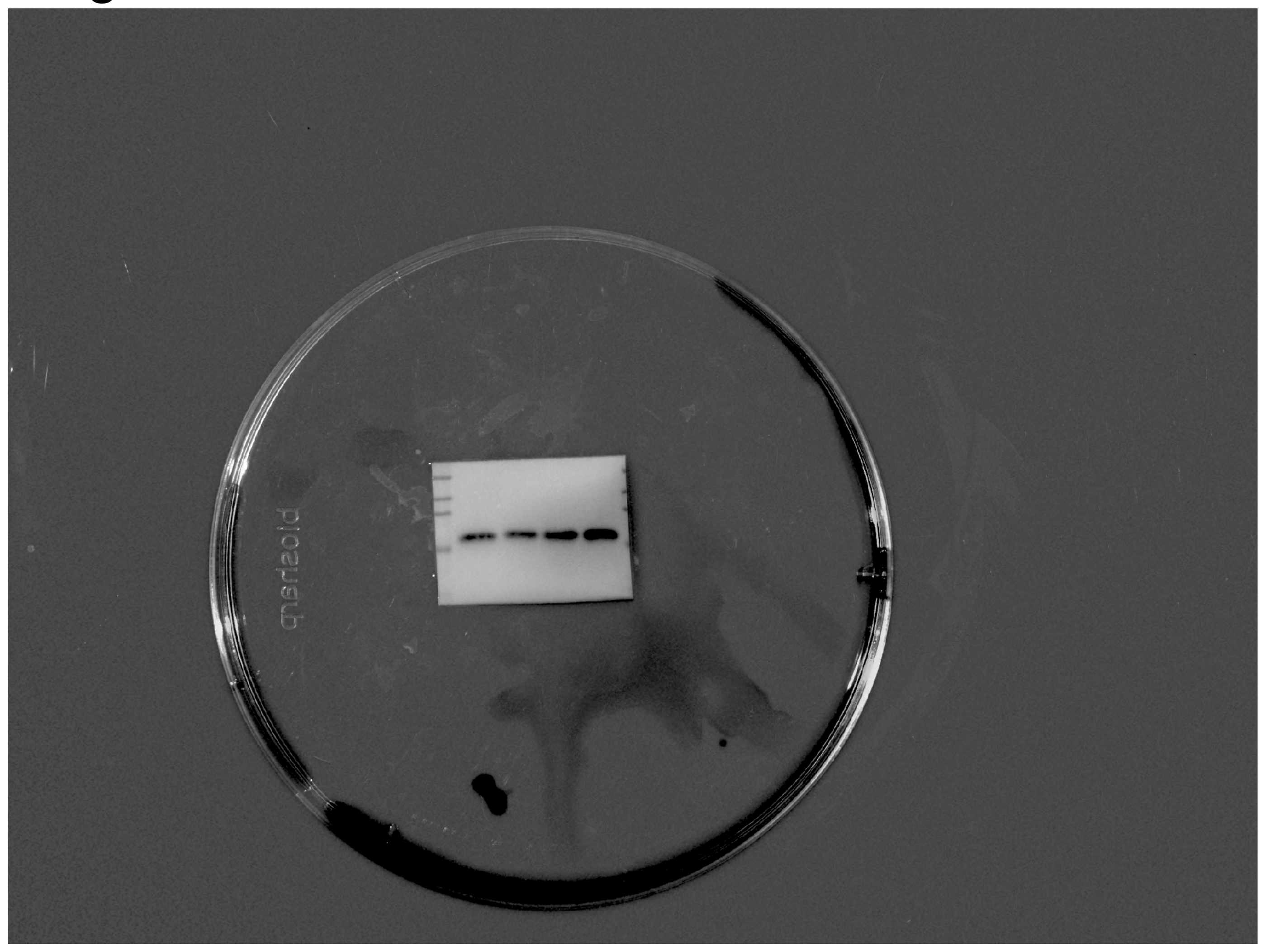

Figure S4A p-4EBP1

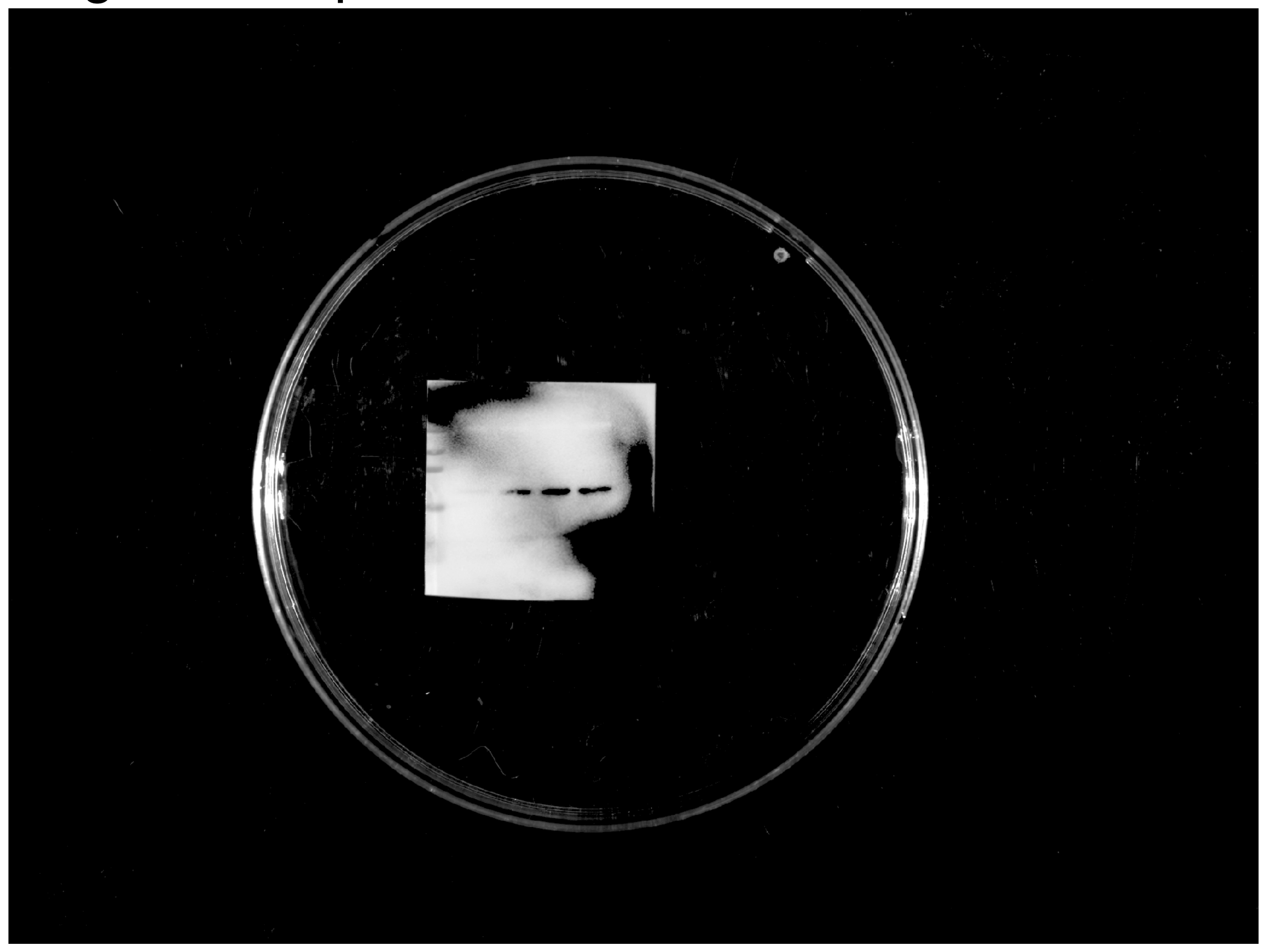

Figure S4A β-actin

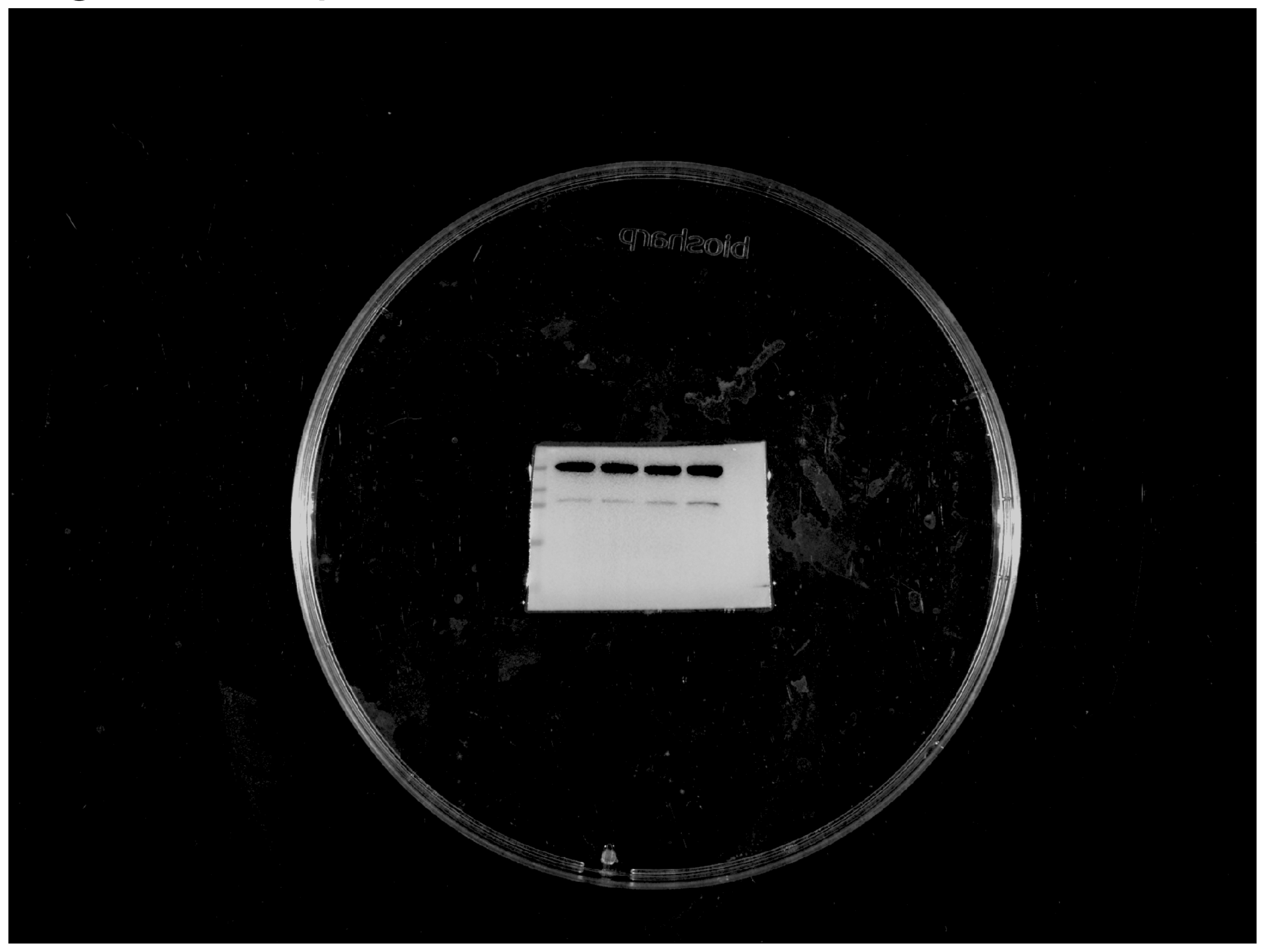

Figure S4C Puro

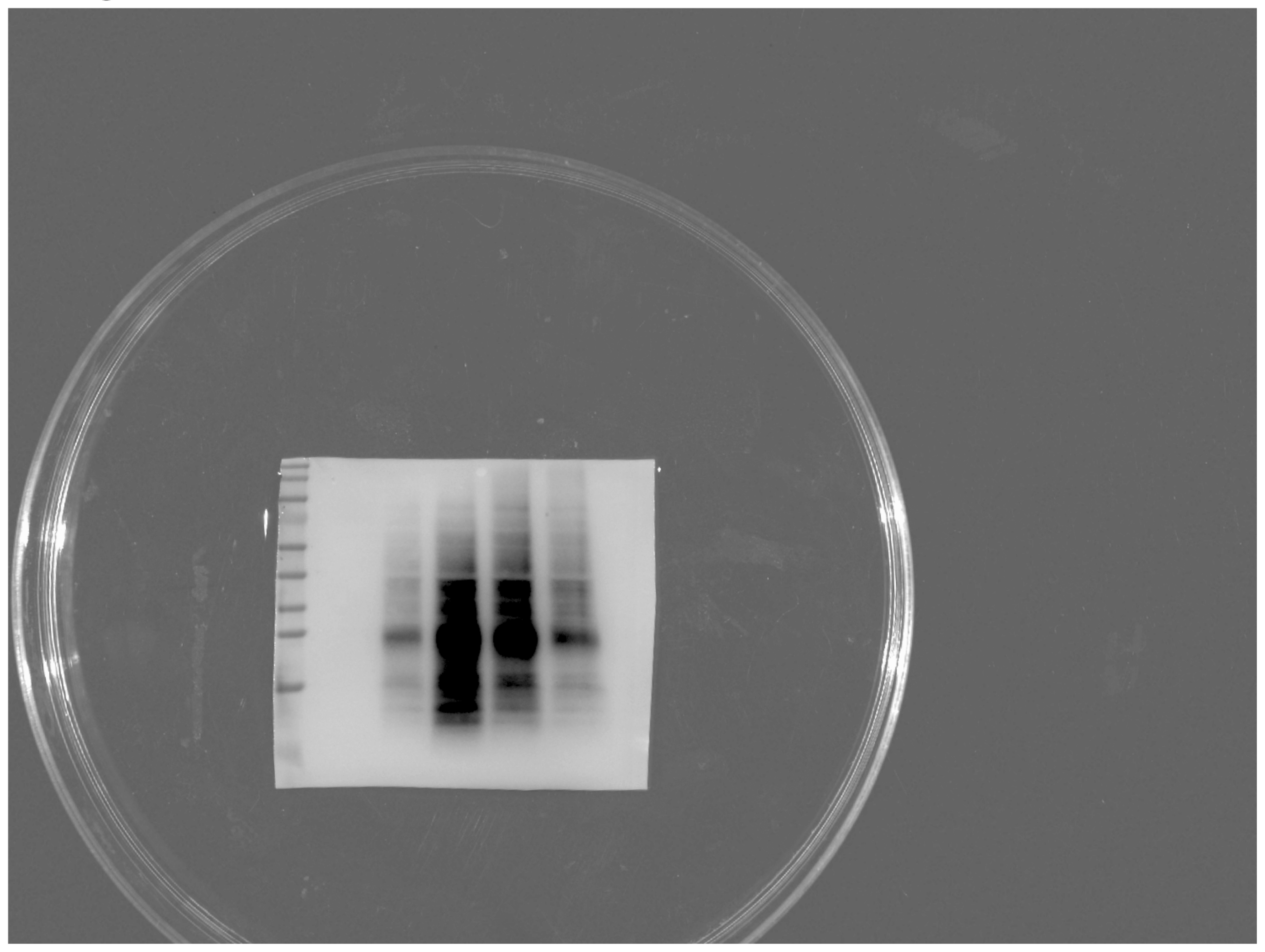

Figure S4C β-actin

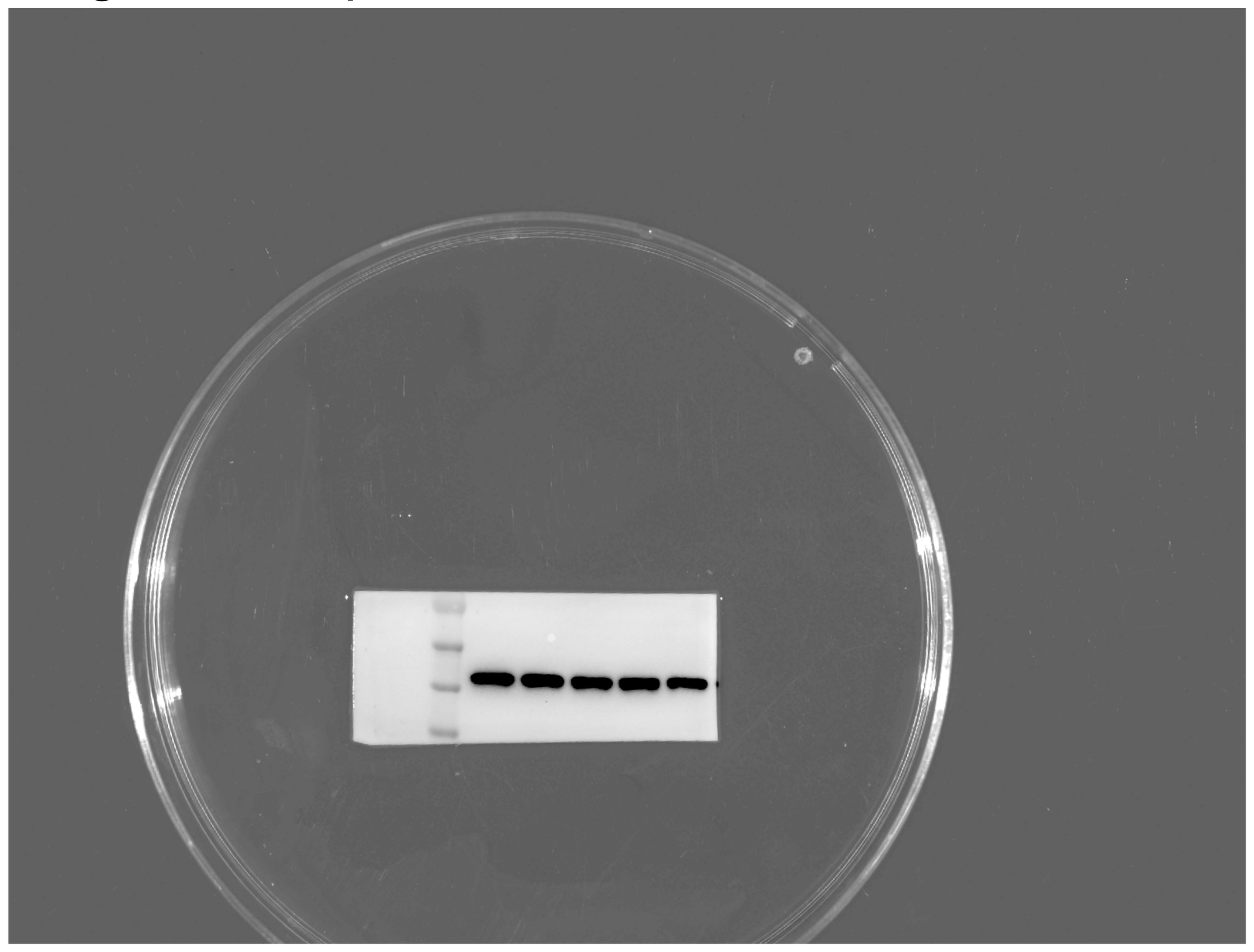

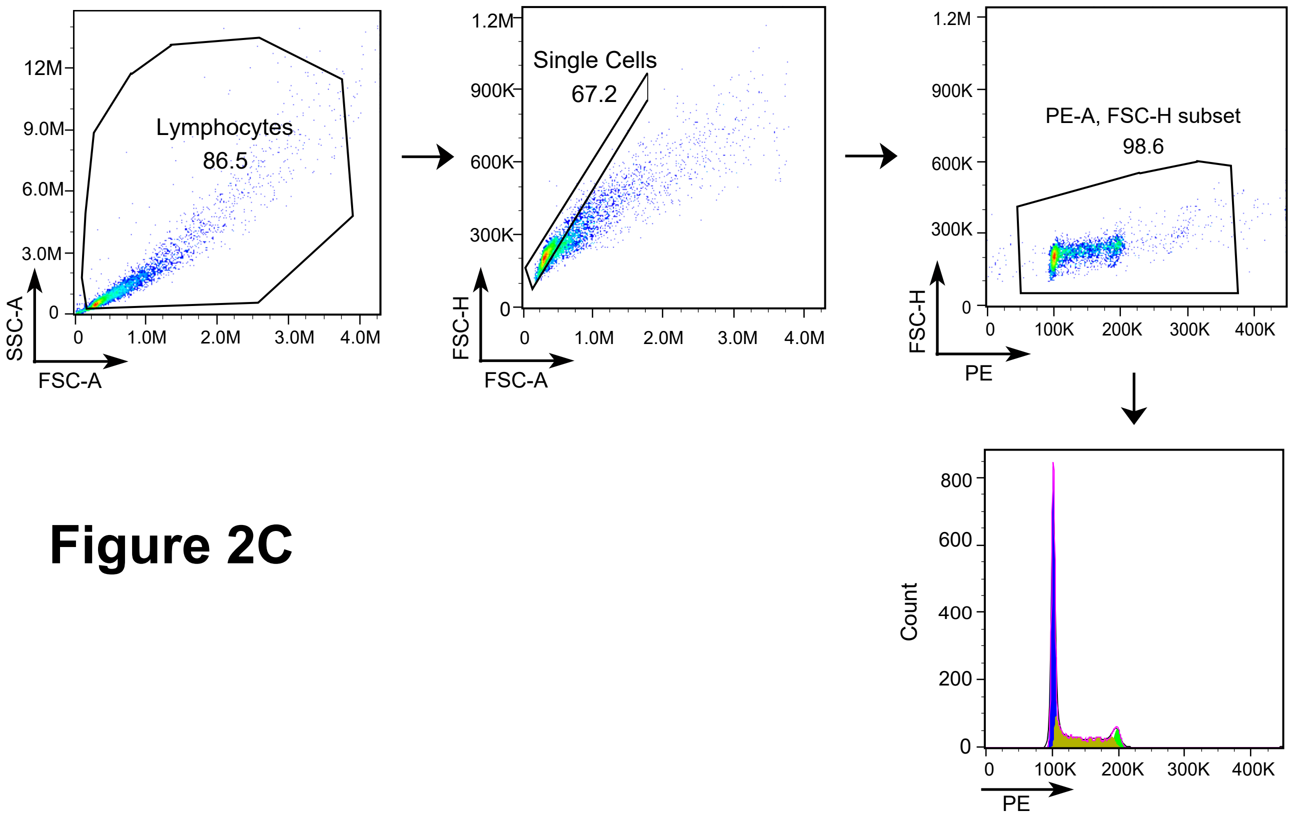

12M
9.0M
6.0M
3.0M
0
SSC-A
0
1.0M
2.0M
3.0M
4.0M
FSC-A
Lymphocytes
86.5
1.2M
900K
600K
300K
0
FSC-H
FSC-A
Single Cells
67.2
1.2M
900K
600K
300K
0
FSC-H
0
100K
200K
300K
400K
PE
PE-A, FSC-H subset
98.6
800
600
400
200
0
Count
PE

Figure 2C
